# Recent Advances in Design, Synthesis, and Biological Activity Studies of 1,3-Selenazoles

**DOI:** 10.3390/biom14121546

**Published:** 2024-12-02

**Authors:** Nataliya A. Makhaeva, Svetlana V. Amosova, Andrey S. Filippov, Vladimir A. Potapov, Maxim V. Musalov

**Affiliations:** A. E. Favorsky Irkutsk Institute of Chemistry, Siberian Branch of the Russian Academy of Sciences, 1 Favorsky Str., Irkutsk 664033, Russia; ggm@irioch.irk.ru (N.A.M.); amosova@irioch.irk.ru (S.V.A.); filippov@irioch.irk.ru (A.S.F.); v.a.potapov@mail.ru (V.A.P.)

**Keywords:** heterocycles, 1,3-selenazoles, synthesis, biological activity, 1,3-selenazole derivatives

## Abstract

The review examines recent advances in the design and synthesis of 1,3-selenazole derivatives since 2000. Various synthetic approaches to 1,3-selenazoles and reaction conditions are discussed. The beneficial properties of 1,3-selenazoles, especially their biological activity, are emphasized. Compounds with antitumor, antiviral (HIV-1 and HIV-2), antibacterial, antifungal, antiproliferative, anticonvulsant, and antioxidant activity are highlighted.

## 1. Introduction

A powerful impulse for the development of organoselenium chemistry came with the discovery of important biological properties of selenium as an essential trace element for animals and humans [1,2,3]. An important role in the body is played by the glutathione peroxidise enzyme, which contains the selenium atom as an active center. A number of organoselenium compounds exhibit high glutathione-peroxidase-like activity [4,5,6].

Over the past two decades, a number of important areas of organoselenium chemistry, including the synthesis and properties of 1,3-selenazole derivatives, have been developing quite intensively. The 1,3-selenazoles derivatives exhibit various biological activities, including antiviral [7,8], antibacterial [9], anticancer [10,11,12], antiparasitic [13], neuroprotective [14], and inhibitory activity to xanthine oxidase [15].

**Figure 1:**
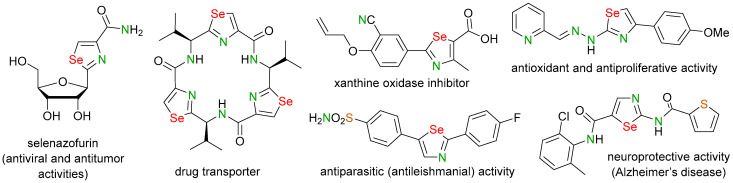
1,3-Selenazoles with biological activity.

Selenazofurin, one of the best known organoselenium compounds, is the 1,3-selenazole derivative that exhibits high antitumor and antiviral properties (Figure 1) [16,17,18,19,20]. Selenazofurin is active against a broad spectrum of DNA and RNA viruses, including both influenza A and B viruses, and it is significantly more potent than its sulfur analogue, the antiviral drug thiazofurin. Selenazofurin is considered to be the first potent selenium-containing antiviral agent that may find medical use [16].

Cyclopeptides containing 1,3-selenazole units are known as promising biologically and pharmaceutically active compounds (Figure 1) [21,22,23,24]. Selenazole derivatives are considered as potential detectors for DNA [25], nucleic acids [26], and proteins [27,28,29,30]. In particular, polymethine cyanine dyes based on 1,3-selenazole derivatives [31,32], as well as polymethine benzoselenazole dye with an electron-withdrawing group at the nitrogen atom of the heterocycles [33], form stabile DNA and dsDNA-complexes, which makes these dyes appropriate for the quantitation and visualization of nucleic acids.

The development of the synthesis and study of the properties of various novel organochalcogen heterocyclic compounds, especially Se,N-containing heterocycles, is within the field of our scientific interests [34,35,36,37,38]. The efficient syntheses of a number of condensed 1,3-selenazole derivatives have been developed [35,36,37,38]. Among these derivatives, compounds with high antibacterial activity have been found. The comparison of the antibacterial properties of condensed 1,3-selenazole derivatives with their sulfur analogs (condensed 1,3-thiazoles) has shown higher activity of the Se/N compounds [38].

Several reviews have recently been published on the synthesis and biological activity of various Se,N-containing heterocyclic compounds [39,40,41,42,43,44].

**Figure 2:**
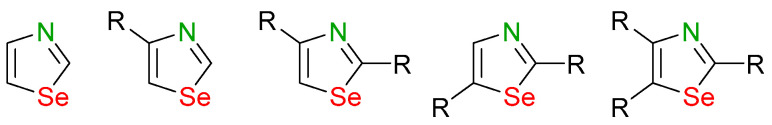
1,3-Selenazoles considered in this review.

This review covers design, synthesis, and biological activity studies on 1,3-selenazoles (Figure 2) and the application of these compounds as valuable intermediates in organic synthesis since 2000.

## 2. 1,3-Selenazole Derivatives

### 2.1. 1,3-Selenazole and 4-Substituted 1,3-Selenazoles

**Scheme 1:**
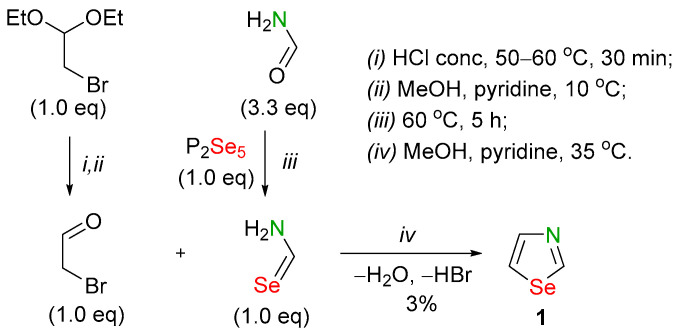
Synthesis of parent 1,3-selenazoles **1**.

Parent 1,3-selenazole **1** was obtained in a 3% yield by the cyclization of selenoformamide with *α*-bromoacetaldehyde (Scheme 1) [45,46]. Selenoformamide was prepared from freshly prepared P_2_Se_5_ and formamide and used in crude form [47].

**Scheme 2:**
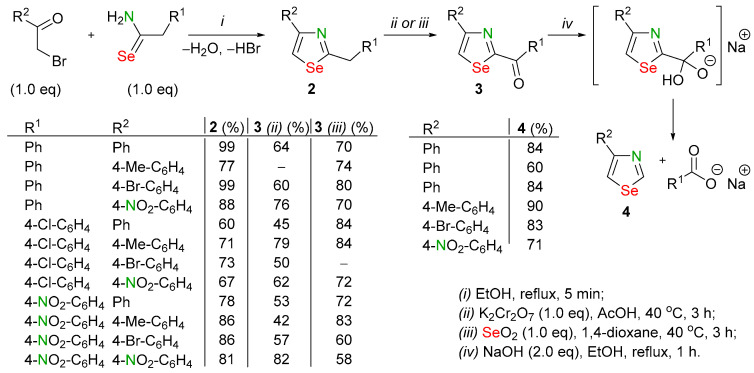
Synthesis of 2-unsubstituted 1,3-selenazoles **4**.

An effective method of the synthesis of 2-unsubstituted 1,3-selenazoles based on 2-benzoyl-1,3-selenazoles was developed (Scheme 2) [48,49]. The cyclization of *α*-bromoketones with selenoamides, prepared from arylacetonitriles and P_2_Se_5_ [47], afforded 2-aryl-1,3-selenazoles **2**, containing the benzyl-, *p*-chlorobenzyl-, and *p*-nitrobenzyl groups, in 60–99% yields. The oxidation of products with K_2_Cr_2_O_7_ or with SeO_2_ in 1,4-dioxane yielded 2-benzoyl-1,3-selenazoles **3** in 45–82% and 70–84% yields, respectively. The treatment of 2-benzoyl-1,3-selenazoles **3** with NaOH in ethanol afforded the desired selenazoles **4** in 60–90% yields [48,49].

**Scheme 3:**
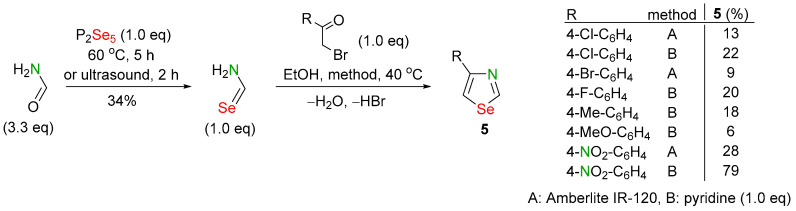
Synthesis of 2-unsubstituted 1,3-selenazoles **5**.

The cyclization of *α*-bromoketones with selenoformamide provided a useful approach to producing 2-unsubstituted 1,3-selenazoles. For example, the reaction of phenacyl bromide with selenoformamide afforded 4-(4-nitrophenyl)-1,3-selenazole (**5**) in a 79% yield. In this case, only two rather than four synthetic stages were required. Obtained 1,3-selenazoles **5** have been shown to have considerable pharmacological significance (Scheme 3) [45,46,48].

### 2.2. 2,4-Substituted 1,3-Selenazoles

**Scheme 4:**
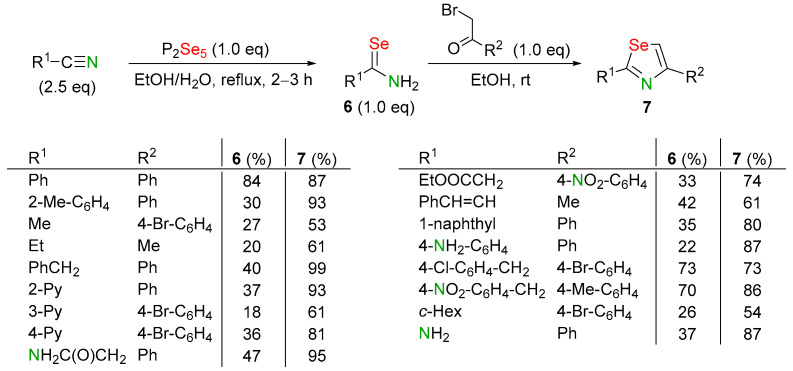
Synthesis of selenoamides **6** and 1,3-selenazoles **7**.

Various 1,3-selenazoles **7** were obtained on the basis of nitriles and freshly prepared P_2_Se_5_. The reaction of nitriles with P_2_Se_5_ in a refluxing EtOH/H_2_O mixture afforded a chemoselective and convenient synthesis of a variety of selenoamides **6**, (18–84% yields) including a number of new functionalized derivatives. Selenoamides are useful building blocks for the synthesis of 1,3-selenazoles and other pharmacologically significant heterocycles containing nitrogen and selenium. The resulting selenoamides **6** were converted into 1,3-selenazoles **7** in 53–99% yields by the reaction with *α*-bromoketones in ethanol solution at room temperature (Scheme 4) [47].

**Scheme 5:**
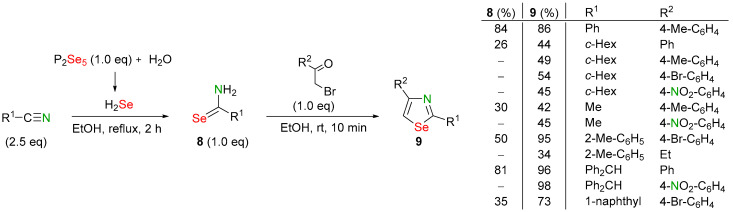
Synthesis of functionalized 1,3-selenazoles **9**.

The reaction of benzonitrile, cyclohexanecarbonitrile, 2-tolylnitrile, acetonitrile, diphenylacetonitrile, and 1-naphthonitrile with P_2_Se_5_ afforded the selenoamides **8** in 26–84% yields. At the same time, the slow adding of water to the reaction mixture resulted in the formation of small amounts of H_2_Se, which subsequently added to the nitriles. The cyclization of selenoamides **8** with phenacyl bromides afforded the functionalized 1,3-selenazoles **9** in 34–98% yield. The yields of the products are generally higher for the aromatic substrates compared to aliphatic compounds (Scheme 5) [50].

**Scheme 6:**
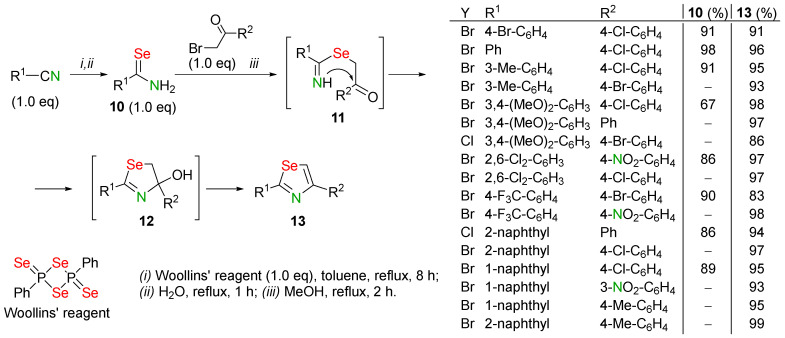
Synthesis of 2,4-diaryl-1,3-selenazoles **13**.

2,4-Diaryl-1,3-selenazoles **13** were prepared by the two-component cyclization of the primary selenoamides with *α*-haloketones (Scheme 6) [51]. Selenoamides **10** were obtained in 86–98% yields by the reaction of Woollins’ reagent with aromatic nitriles in refluxing toluene, followed by hydrolysis with water. A solution of *α*-haloketone in MeOH was added to a refluxing solution of arylselenocarboamide in MeOH and the reaction mixture was then refluxed for 1 h. A series of 2,4-diaryl-1,3-selenazoles **13** was prepared in 86–99% yields based on this approach. The reaction proceeded with the formation of intermediate **11** followed by the intramolecular cyclization and the dehydration process of intermediate **12**.

**Scheme 7:**
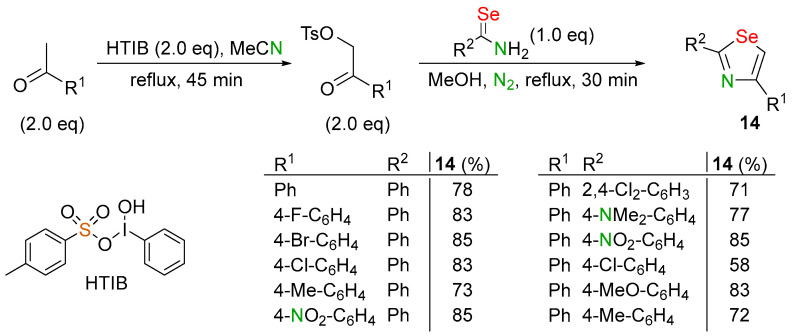
Synthesis of 2,4-substituted 1,3-selenazoles **14** with using HTIB.

A versatile reagent for organic synthesis, [hydroxy(tosyloxy)iodo]benzene (HTIB), is especially useful in the reaction with ketones. The tosyloxylation of ketones with HTIB, followed by the treatment with primary selenoamides, provides a convenient method of synthesis for 1,3-selenazoles, avoiding the use of toxic and lachrymatory *α*-haloketones. Thus, 2,4-substituted 1,3-selenazoles **14** were prepared in 58-85% yields from the appropriate ketones and selenoamides under reflux in a solvent mixture MeCN/MeOH (Scheme 7) [52].

**Scheme 8:**
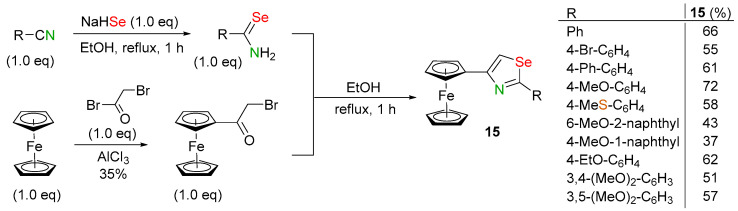
Synthesis of 2-aryl-4-ferrocenyl-1,3-selenazoles **15**.

New 2-aryl-4-ferrocenyl-1,3-selenazoles **15** were synthesized in 43–72% yields by the reaction of aryl selenoamides with (2-bromoacetyl)ferrocene in refluxing ethanol. Aryl selenoamides were prepared by the treatment of aromatic nitriles with NaHSe in refluxing ethanol. 2-bromoacetyl ferrocene was prepared in 35% yield by the reaction of ferrocene with bromoacetyl bromide in the presence of AlCl_3_ (Scheme 8) [53].

The obtained products (**15)** exhibit high antibacterial activity against *Escherichia coli, Staphylococcus aureus,* and *Pseudomonas aeruginosa* [53].

**Scheme 9:**
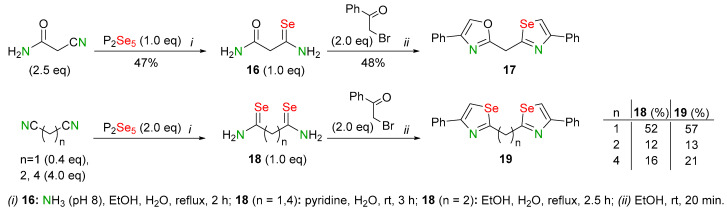
Synthesis of functionalized 1,3-selenazoles **17** and **19**.

The reaction of cyanoacetamide with P_2_Se_5_ afforded malonic selenoamide **16** in 47% yield. The reaction of the product **16** with two equivalents of phenacyl bromide resulted in the formation of (4’-phenyloxazolyl-2’-yl)-4-phenylselenazolyl-2-methane (**17**) in 48% yield, containing the oxazole and selenazole rings (Scheme 9) [50].

Moreover, the use of P_2_Se_5_ allowed researchers to carry out the convenient synthesis of selenocarboxylic diamides, which were converted into bis(selenazol-2-yl)alkanes. The reaction of P_2_Se_5_ with malonic dinitrile, 1,2-dicyanoethane, and 1,4-dicyanobutane yielded malonic diselenoamide, succinic diselenoamide, and adipic diselenoamide (**18**). The double cyclization of compounds (**18**) with phenacyl bromide in the EtOH/H_2_O mixture afforded bis-selenazoles **19** (Scheme 9) [50].

Reactions of 1,3-dicyanobenzene with a potassium 4-methylbenzenecarboselenoate afforded 3-cyanobenzenecarboselenoamide **20** and 1,3-benzenedicarboselenoamide **21** in 49% and 24% yields, respectively. 

**Scheme 10:**
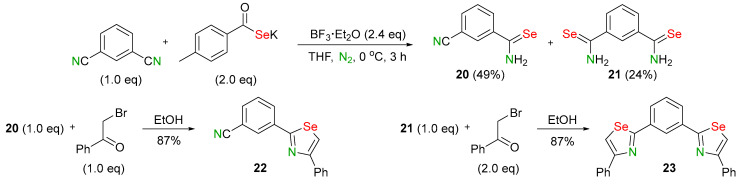
Synthesis of 2,4-functionalized 1,3-selenazole **22** and bis-1,3-selenazole **23**.

The reaction was carried out under an argon atmosphere at 0 °C in THF in the presence of boron trifluoride ether complex. After workup, the residue was purified by flash chromatography. Prepared 3-cyanobenzenecarboselenoamide **20** and 1,3-benzenedicarboselenoamide **21** were used as substrates for the preparation of 1,3-selenazoles. Selenazole **22** and diselenazole **23** were obtained in 87% yields by the reaction of selenoamides **20** and **21** with phenacyl bromide in EtOH (Scheme 10) [54].

**Scheme 11:**
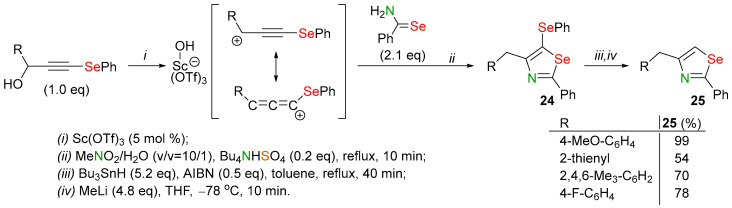
Synthesis of 4-arylmethyl-2-phenyl-1,3-selenazoles **25**.

A new cycloaddition reaction of the phenylselanylpropargyl alcohols with selenobenzamide in the presence of catalytic system Sc(OTf)_3_-MeNO_2_-H_2_O-Bu_4_NHSO_4_ was developed (Scheme 11) [55]. The use of this effective catalytic system allowed researchers to generate *α*-selanylpropadienyl cations, which were involved into cycloaddition reactions to form cycloadducts **24**. 4-arylmethyl-2-phenyl-1,3-selenazoles **25** were obtained in 54–99% by the treatment of cycloadducts **24** with tributyltin hydride in the presence of azobisisobutyronitrile (AIBN) and methyllithium (Scheme 11) [55].

**Scheme 12:**
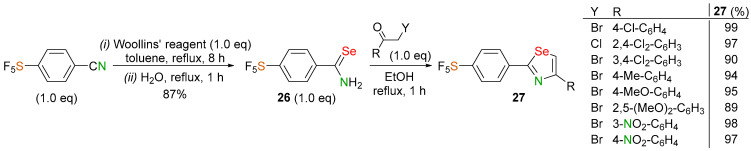
Synthesis of 2,4-diaryl-1,3-selenazoles **27**.

A number of novel 2,4-diaryl-1,3-selenazoles **27** bearing pentafluorosulfanyl SF_5_ functional groups were obtained by the two-component cyclization of selenoamide with *α*-haloketones (Scheme 12) [56]. The initial selenoamide **26** was obtained in an 87% yield from the reaction of Woollins’ reagent with 4-pentafluorosulfanylbenzonitrile, followed by hydrolysis. The cyclization of selenoamide **26** with *α*-haloketones in refluxing ethanol made it possible to obtain a number of 1,3-selenazoles **27** in 89–99% yields (Scheme 12) [56].

**Scheme 13:**
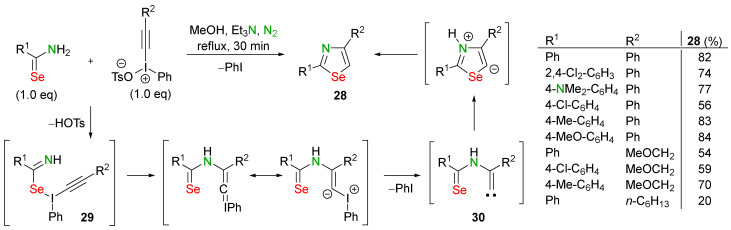
Synthesis of 2,4-substituted 1,3-selenazoles **28** using alkynyl(phenyl)iodonium salts.

A method for the synthesis of 2,4-substituted 1,3-selenazoles **28** in 20–84% yields by the cyclocondensation of primary selenoamides and alkynyl(phenyl)iodonium salts was developed (Scheme 13) [57]. The synthesis was carried out in the presence of Et_3_N in MeOH under a nitrogen atmosphere.

A probable mechanism for the formation of selenazoles **28** includes the selenophilic attack of the iodonium atom of alkynyl(phenyl)iodonium salts on the selenoamides with the formation of primary adducts **29**, followed by an uncommon polyhetero-Claisen rearrangement and 1,1-elimination of iodobenzene with the formation of carbene **30**. This synthetic method is simple and mild and provides good yields of the products (**28**) (Scheme 13) [57].

**Scheme 14:**
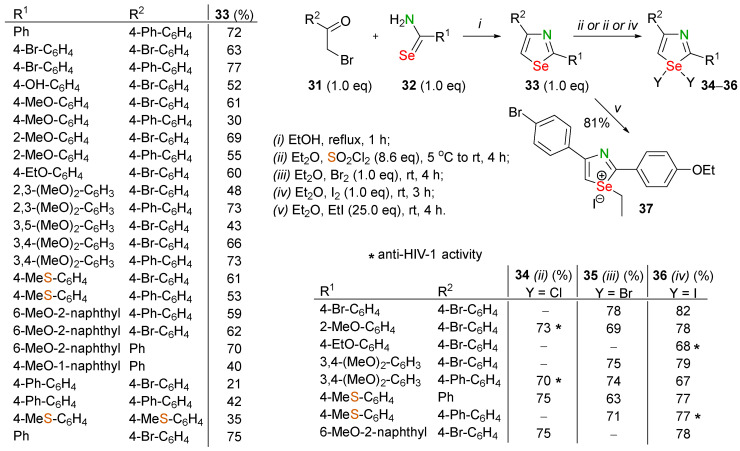
Synthesis of 1,1-dihalo-2,4-diaryl-1,3-selenazoles **34–36** and selenonium salt **37**.

The cyclization of primary arylselenoamides **32** with *α*-bromoketones **31** in EtOH afforded a variety of 2,4-diaryl-1,3-selenazoles **33** in 21–77% yields (Scheme 14) [58]. The halogenation of the 2,4-diaryl-1,3-selenazoles **33** with SO_2_Cl_2_, bromine and iodine in diethyl ether yielded 1,1-dihalo-2,4-diaryl-1,3-selenazoles **34**–**36** in good yields (63–82%) while the reaction of compound **33** (R^1^ = 4-EtO-C_6_H_4_, R^2^ = 4-Br-C_6_H_4_) with ethyl iodide yielded selenonium salt **37** in an 81% yield (Scheme 14) [58].

The antiviral activity of 1,1-dihalo-2,4-diaryl-1,3-selenazoles **34** and **36** was investigated against the AIDS virus (HIV-1, HIV-2). It was found that these compounds exhibit antiviral activity against HIV-1 [58].

**Scheme 15:**
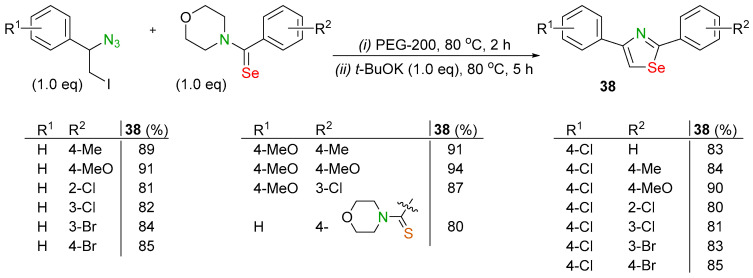
Synthesis of 2,4-aryl-substituted 1,3-selenazole derivatives **38**.

A practical strategy to access diversified 3-selenazole scaffolds was designed. Aryliodoazides were used as the versatile reagent; these can react with a wide range of selenoamides. The reaction of iodoazides with selenoamides proceeded efficiently using *t*-BuOK as the base in PEG-200 as a nontoxic solvent to obtain the target products (**38**) in 80–91% yields (Scheme 15). The special characteristics of this protocol are its ease of operation and high functional group tolerance, making it a practical and convenient route for the preparation of various libraries of 1,3-selenazoles (Scheme 15) [59].

**Scheme 16:**
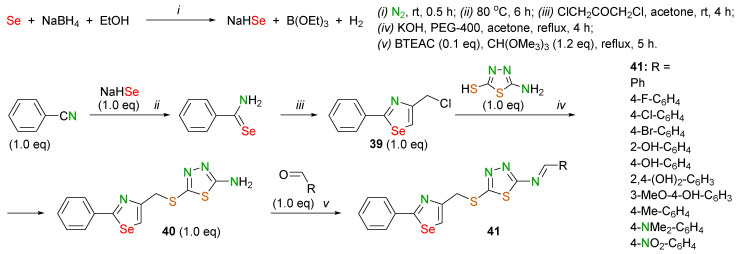
Synthesis of 1,3-selenazole-containing 1,3,4-thiadiazole derivatives **41**.

A number of 1,3,4-thiadiazole derivatives containing 1,3-selenazole with fragments of Schiff base were synthesized (Scheme 16) [60]. As a result of the interaction of benzonitrile with NaHSe, selenobenzamide was obtained, and this reacted with 1,3-dichloroacetone in acetone at room temperature to form compound **39**. Product **40** was obtained by the reaction of 1,3-selenazole **39** and 2-amino-5-mercapto-1,3,4-thiadiazole using PEG-400 as a catalyst under reflux. Finally, the condensation of compound **40** and substituted aromatic aldehydes in the presence of trimethyl orthoformate using benzyl triethylammonium chloride (BTEAC) as a catalyst made it possible to obtain the target compounds **41** under reflux. As a result of this reaction, a series of 1,3,4-thiadiazole derivatives containing 1,3-selenazole **41** with Schiff base fragments were prepared in 38–72% yields. These derivatives have an increased number of active groups in the molecule (Scheme 16) [60].

The majority of the obtained compounds exhibited high antiproliferative activity against mouse lymphocyte leukemia cells (L1210) and human breast cancer cells (MCF-7). In particular, compound **41** (R = 4-Cl-C_6_H_4_) demonstrated the most potent antiproliferative activity against MCF-7 cells compared to 2-phenyl-4-carboxyl-1,3-selenazole (PCS), which was used as a control compound [60].

**Scheme 17:**
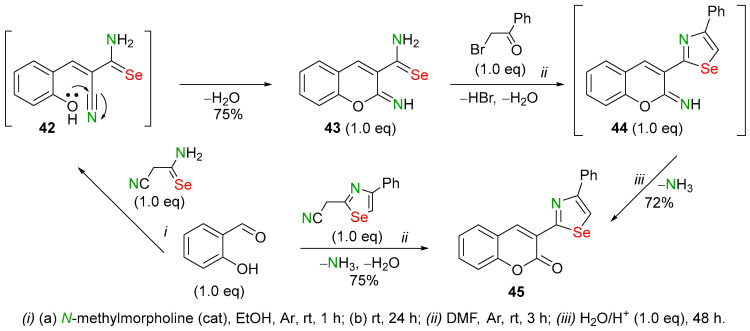
Synthesis of 3-(4-phenyl-1,3-selenazol-2-yl)-2*H*-chromen-2-one **45**.

The convenient synthesis of a chromenone derivative, bearing the 1,3-selenazole ring, was developed (Scheme 17) [61]. The condensation of salicylaldehyde with cyanoselenoacetamide under Knoevenagel reaction conditions led to the formation of intermediate **42**. Subsequent pyran ring closure proceeded to yield 2-oxo-2*H*-chromeno-3-carboselen-amide **43** in a 75% yield. The product **43** entered easily into the Hantzsch-type condensation with phenacyl bromide in DMF at room temperature to form 3-selenazolyl-substituted 2-iminocoumarin **44**, which was rapidly hydrolyzed under the reaction conditions to form 3-(4-phenyl-1,3-selenazol-2-yl)-2*H*-chromen-2-one **45** in a 72% yield. Compound **45** was also obtained in a 75% yield by the condensation of salicylaldehyde with (4-phenyl-1,3-selenazol-2-yl)acetonitrile (Scheme 17) [61].

**Scheme 18:**
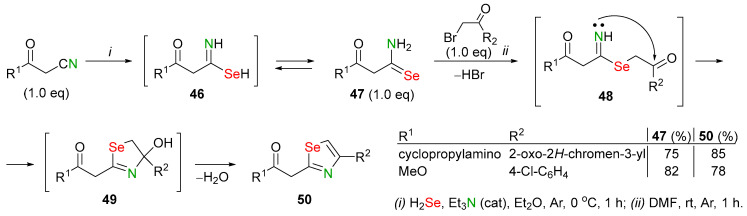
Synthesis of functionally substituted 1,3-selenazoles **50**.

2,4-Subtituted 1,3-selenazoles were synthesized using new reagents, 3-amino-*N*-cyclopropyl-3-selanylidene-propanamide and methyl 3-amino-3-selanylidenepropanoate **47** (Scheme 18) [62]. The derivatives of 2-(carbamoselenoyl)acetic acid **47** were obtained by the reaction of 2-cyano-*N*-cyclopropylacetamide and methyl 2-cyanoacetate with H_2_Se in diethyl ether at 0 °C under argon in the presence of triethylamine. Compounds (**47)** were converted to functionally substituted 1,3-selenazoles **50** by reaction with *α*-bromoketones in DMF at room temperature under argon (Scheme 18) [62].

A plausible reaction pathway is proposed [62]. At the initial stage, the hydroselenide ion is generated from H_2_Se and adds to the cyano group of starting cyanides to yield intermediate **46**, which is stabilized as selenoamides **47**. The reaction of selenoamides **47** with *α*-bromoketones leads to selenide **48**, which undergoes intramolecular cyclization to substituted dihydroselenazole **49**. The latter undergoes dehydration to afford final aromatic 1,3-selenazoles **50** (Scheme 18) [62].

**Scheme 19:**
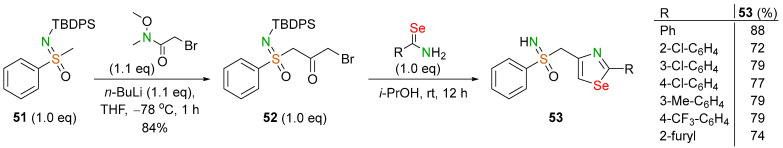
Synthesis of sulfoximine tethered 2,4-substituted 1,3-selenazoles **53**.

A metal-free method was efficiently developed for the synthesis of sulfoximine tethered selenazoles. *N*-TBDPS-protected sulfoximine **51** was treated with *n*-BuLi followed by the addition of 2-bromo-*N*-methoxy-*N*-methylacetamide (Scheme 19) [63]. A key compound, *α*-bromoalkanone **52**, was obtained in an 84% yield. The target functionalized selenazoles **53** were synthesized in good yields (72–88%) by the reacting compound **52** and the selenoamides in 2-propanol at an ambient temperature (Scheme 19) [63]. The cyclization and the TBDPS deprotection processes occurred as a one-pot procedure. The developed metal-free approach to 2,4-substituted 1,3-selenazoles **53** can be considered a very useful contribution to the growing field of the synthesis and studies of sulfoximine compounds in medicinal chemistry.

**Scheme 20:**
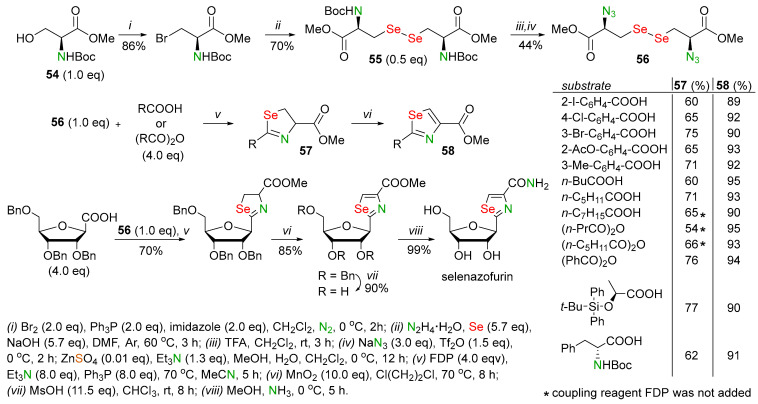
Synthesis of 2,4-disubstituted 1,3-selenazoles **58** and selenazofurin.

A novel method for the one-pot cascade synthesis of 2,4-disubstituted 1,3-selenazoles was developed (Scheme 20) [64]. Triethylamine, pentafluorophenyl diphenylphosphate (FDP), *β*-azido diselenide **56,** and triphenylphosphine were added to a solution of acid or anhydride in acetonitrile and the reaction mixture was stirred at 70 °C. This approach allowed obtaining selenazolines **57** in 54–77% yields. In turn, selenazolines **57** were oxidized by activated manganese oxide in 1,2-dichloroethane to yield selenazoles **58** in 89–95% yields (Scheme 20) [64]. Azido diselenide **56** was obtained in a 44% yield from commercially accessible *N*-Boc-*L*-serine methyl ester (**54**) in several stages with the formation of the intermediate *N*-Boc-protected homoselenocystine **55**. The valuable application of this method could be seen in the efficient total synthesis of selenazofurin, which showed high antitumor activities (Scheme 20) [64].

**Scheme 21:**
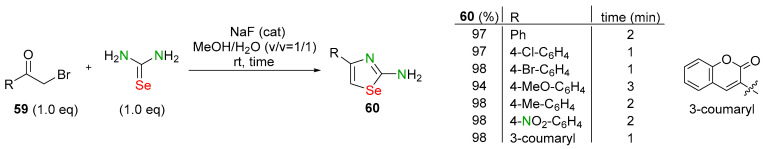
Synthesis of substituted 1,3-selenazole derivatives **60** catalyzed by NaF.

Sodium fluoride has been found to be a mild, simple, and efficient catalyst for the synthesis of 2,4-disubstituted 1,3-selenazoles using phenacyl bromides and 3-(2-bromoacetyl)-2*H*-chromen-2-one with selenourea in aqueous MeOH at an ambient temperature (Scheme 21) [65]. Presumably, the fluoride ion acts as a base in this reaction.

Sodium fluoride was added to the solution of the corresponding phenacylbromide or 3-(2-bromoacetyl)-2*H*-chromen-2-one **59** and selenourea in MeOH. After stirring the mixture at an ambient temperature for 1-3 min, water was added and analytically pure substituted 1,3-selenazole derivatives **60** were separated in excellent yields as solids (Scheme 21) [65].

**Scheme 22:**
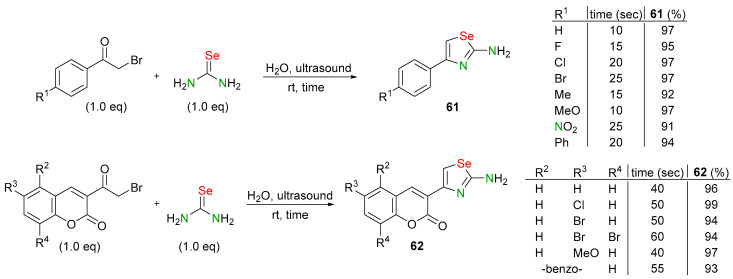
Synthesis of 2,4-disubstituted-1,3-selenazoles **61,62** under ultrasonic irradiation.

A rapid and efficient procedure for the synthesis of 2,4-disubstituted-1,3-selenazoles **61,62** was developed based on the reaction of *α*-bromoketones with selenourea at room temperature in aqueous medium under ultrasonic irradiation. Analytically pure products **61,62** were formed in 91–99% yields for 10–60 s. The advantages of this methodology include its operational simplicity, ability to save time, use of mild reaction conditions, and lack of by-products (Scheme 22) [66].

**Scheme 23:**
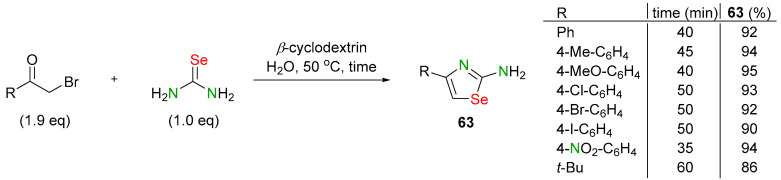
Synthesis of 2-amino-4-substituted-1,3-selenazoles **63**.

2-amino-4-substituted-1,3-selenazoles **63** were synthesized in 86–95% yields from *α*-bromoketones and selenourea in the presence of *β*-cyclodextrin in water at 50 °C under atmospheric pressure. This methodology also avoids an inert atmosphere and high temperatures, making it more convenient to use (Scheme 23) [67].

**Scheme 24:**
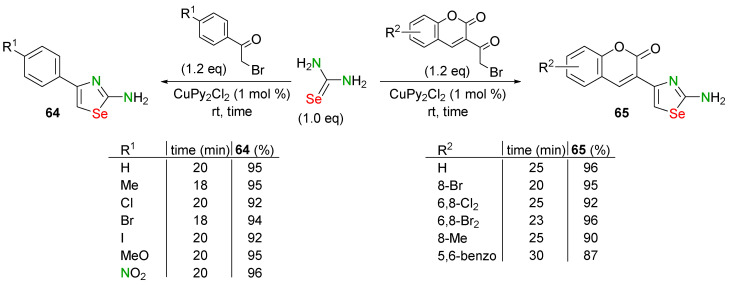
Synthesis of 2-amino-1,3-selenazoles **64,65** in the presence of CuPy_2_Cl_2_.

Bromo acetyl benzene or 3-bromo acetyl coumarin derivatives and selenourea in the presence of CuPy_2_Cl_2_ reacted very efficiently in a mortar without any solvent. CuPy_2_Cl_2_ was added to a mixture of *α*-bromoketone and selenourea in a mortar and ground with a pestle at an ambient temperature. The reactions were completed within 30 min of milling, and the required products **64**,**65** were obtained in high yields (87–96%). The reaction under solid-phase conditions was environmentally safe, completed in higher yields, and had a more convenient workup (Scheme 24) [68].

**Scheme 25:**
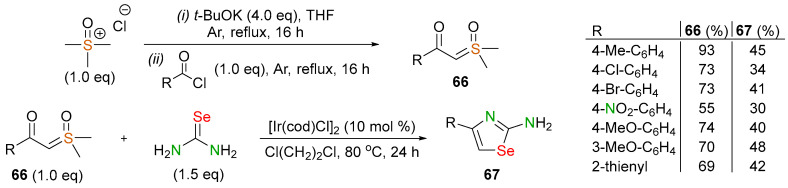
Synthesis of 4-aryl-substituted 2-amino-1,3-selenazoles **67**.

A series of 1,3-selenazoles were synthesized via Ir-catalyzed sulfoxonium ylide insertion chemistry (Scheme 25) [69]. Sulfoxonium ylides **66** were synthesized as precursors to 1,3-selenazoles from trimethylsulfoxonium chloride and acid chloride substrate in THF using *t*-BuOK in 55–93% yields. Then, a mixture of *β*-ketosulfoxonium ylide **66** and selenourea in dichloroethane was heated to 80 °C and [Ir(cod)Cl]_2_ was added to the mixture. The resulting mixture was stirred at 80 °C. After workup, 4-aryl-substituted 2-amino-1,3-selenazoles **67** were isolated in 30–48% yields. This was a useful, substrate-tolerant approach to 1,3-selenazoles that may find application in medicinal chemistry (Scheme 25) [69].

**Scheme 26:**
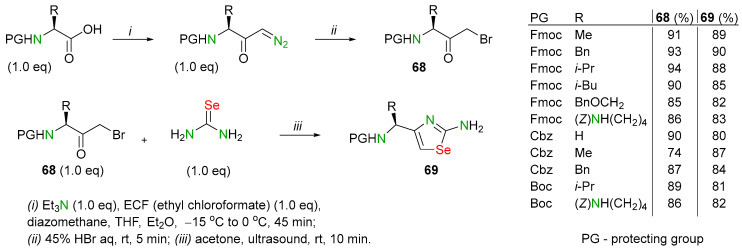
Ultrasound mediated synthesis of 2-amino-1,3-selenazoles **69** obtained from Fmoc/Boc/Z-*α*-amino acids.

An efficient and simple one-pot synthesis of Fmoc/Boc/Z-amino acid derived from 2-amino-1,3-selenazoles **69** was developed (Scheme 26) [70]. At first, *N*-Fmoc/Boc/Z-amino acid was transformed into the corresponding diazomethyl ketone. The conversion of the latter compound into bromomethyl ketone **68** was carried out in the presence of HBr within 10 min in a near-quantitative yield. Then, the 2-amino-1,3-selenazole analogues of Fmoc/Boc/Z-amino acids **69** were synthesized in 80-90% yields upon treating the *N*-protected bromomethyl ketones **68** with selenourea in acetone under ultrasonication (Scheme 26) [70].

**Scheme 27:**
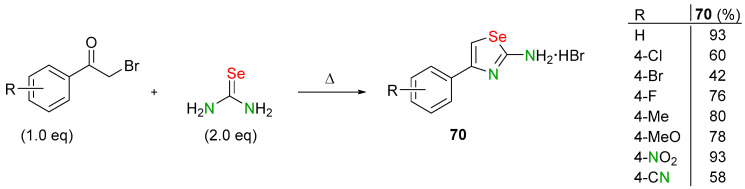
Solvent-free synthesis of 4-aryl-1,3-selenazol-2-amine hydrobromides **70**.

A simple and environmentally tolerant solvent- and catalyst-free protocol for the synthesis of 2-amino-1,3-selenazoles **70** through Hantzsch-type condensation was developed (Scheme 27) [71]. Powdered selenourea was added to 2-bromoacetophenone heated to the melting point. The reactions were completed within a few seconds, and the desired 4-aryl-1,3-selenazol-2-amine hydrobromides **70** were obtained in 42–93% yields (Scheme 27) [71]. This reaction procedure is an example of a promising approach that can be used in green chemistry.

**Scheme 28:**
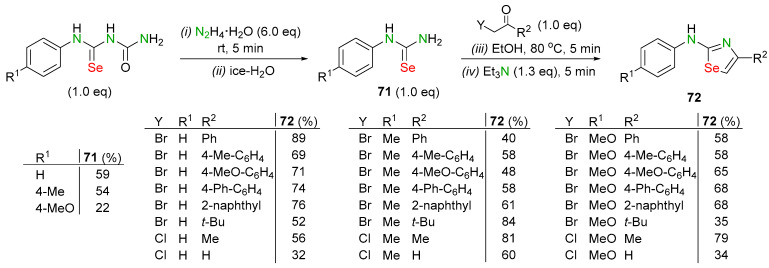
Synthesis of functionalized 2-arylamino-1,3-selenazoles **72**.

A series of functionalized 2-arylamino-1,3-selenazoles **72** were synthesized (Scheme 28) [72]. The starting compounds, *N*-aryl-*N’*-benzoylselenoureas, were treated with an excess of hydrazine hydrate under solvent-free conditions. Upon the addition of cold water, the compounds **71** precipitated and were easily isolated (22–59% yields). The target 2-arylamino-1,3-selenazoles **72** were obtained in 32–89% yields from the cyclocondensation reaction of arylselenoureas **71** with 2-haloketones in the presence of Et_3_N (Scheme 28) [72].

The promising compounds (**72**, R^1^ = H, R^2^ = 4-MeO-C_6_H_4_; R^1^ = H, R^2^ = Me) were found, and these showed relatively low toxicity and high activity against the fungal strains *C. neoformans* and *C. albicans* [72].

**Scheme 29:**
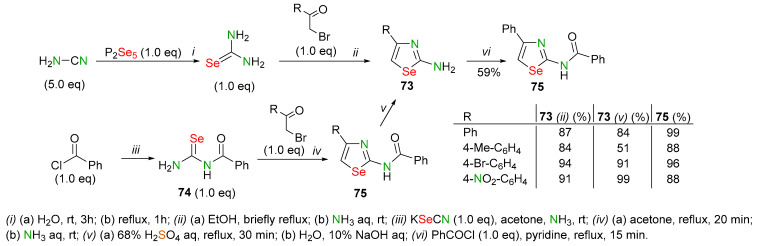
Synthesis of 2-aminoselenazoles **73** and the protected 2-aminoselenazoles **75**.

2-aminoselenazoles **73** were obtained by two alternative methods. On the one hand, the reaction of P_2_Se_5_ with cyanamide yielded selenourea. The cyclization of selenourea with *α*-bromoketones afforded the expected primary 2-aminoselenazoles **73** in a 84–94% yield (Scheme 29) [50].

However, another approach did not require the use of selenourea for the synthesis of 2-aminoselenazoles **73**. The stable benzoylselenourea (**74**) was obtained by the reaction of an acetone solution of potassium selenocyanate with benzoyl chloride and subsequent addition of ammonia. The cyclization of compound **74** with *α*-bromoketones afforded the protected 2-aminoselenazoles **75** (88–99% yields), which were deprotected by the treatment with sulfuric acid, providing products (**73)** in good yields (51–99%) (Scheme 29) [50].

In addition, 2-amino-5-phenyl-1,3-selenazole **73** (R = Ph) was transformed into 2-benzoylamino-4-phenyl-selenazole **75** (59% yield) by refluxing with benzoyl chloride in pyridine solution (Scheme 29) [49].

**Scheme 30:**
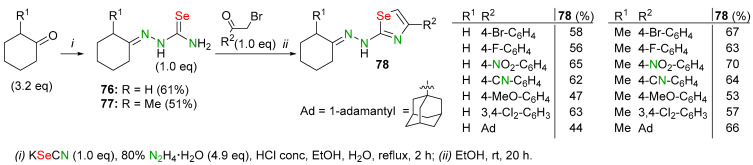
Synthesis of 2,4-disubstituted 1,3-selenazoles **78** with antimicrobial activity.

Fourteen biologically active 2,4-disubstituted 1,3-selenazoles **78** were synthesized (Scheme 30) [73]. At the first stage, selenosemicarbazones **76** and **77** were synthesized by the reaction of commercially available cyclohexanone and 2-methylcyclohexanone with KSeCN and hydrazine hydrate in the presence of HCl. The products **76** and **77** were obtained in 61% and 51% yields, respectively. Next, 1,3-selenazoles **78** were prepared in 44–70% yields and with high chemical and isomeric purity (100% *E*-isomer) by the Hantzsch-type cyclization reaction of cyclohexanone selenosemicarbazone **76** and racemic 2-methylcyclohexanone selenosemicarbazone **77** with appropriate para-substituted bromoacetophenone and 1-adamantyl bromomethyl ketone (Scheme 30) [73].

According to research results, the products with F, NO_2_, CN, and MeO substituents and the cyclohexanone and 2-methylcyclohexanone derivatives exhibited antifungal activity. Compounds with 3,4-dichlorophenyl and adamantanyl substituents also showed antibacterial activity [73].

**Scheme 31:**
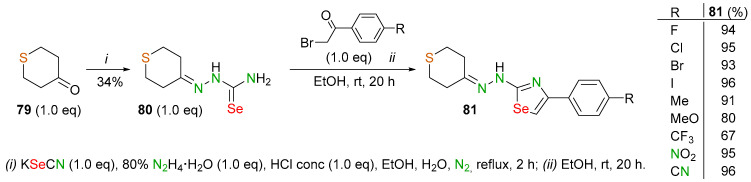
Synthesis of 2,4-disubstituted 1,3-selenazoles **81** based on dihydro-2*H*-thiopyran-4(3*H*)-one (**79**).

Nine 2,4-substituted 1,3-selenazoles based on dihydro-2*H*-thiopyran-4(3*H*)-one (**79**) were prepared. At first, 2-(tetrahydro-4*H*-thiopyran-4-ylidene)hydrazinecarboselenoamide (**80**) was prepared from compound **79** using KSeCN and hydrazine hydrate. The Hantzsch-type condensation of appropriate para-substituted bromoacetophenones with selenosemicarbazone **80** afforded the selenazoles **81** in good yields (67–96%) and with high chemical purity (Scheme 31) [74].

The synthesized compounds exhibited high antifungal activity against *Candida* spp and antibacterial activity against Gram-positive bacteria. In addition, some compounds showed significant anticonvulsant activity [74].

**Scheme 32:**
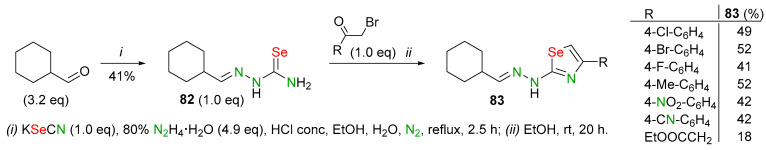
Synthesis of 2,4-disubstituted 1,3-selenazoles **83** based on cyclohexanecarbaldehyde.

The synthesis of 1,3-selenazoles **83** with antimicrobial activities was developed (Scheme 32) [75]. At the first stage, the cyclohexanecarbaldehyde selenosemicarbazone **82** was synthesized from cyclohexanecarbaldehyde.

Then, the Hantzsch-type cyclization reaction of selenosemicarbazone **82** with appropriate para-substituted bromoacetophenones or ethyl 4-chloroacetoacetate under a nitrogen atmosphere afforded 2,4-substituted 1,3-selenazoles **83** in 18–52% yields (Scheme 32) [75].

Compounds (**83)** (except R = EtOOCCH_2_) were found to be active against Gram-positive bacteria, including *Staphylococcus aureus* and pathogenic staphylococci [75].

**Scheme 33:**
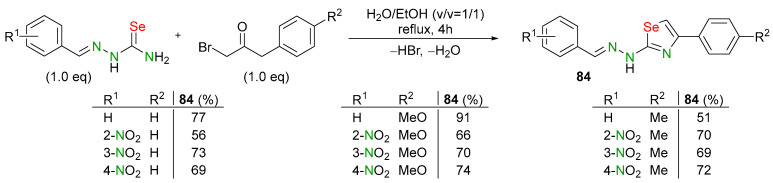
Synthesis of benzylidene-based (1,3-selenazol-2-yl)hydrazones **84**.

Selenazolyl-hydrazones **84** were prepared by the reaction of selenosemicarbazones and *α*-bromocarbonyl derivatives (Scheme 33) [76]. Selenosemicarbazones were dissolved in a mixture of H_2_O and EtOH (v/v=1/1) and *α*-bromocarbonyl derivatives were added. The reaction mixtures were stirred and refluxed. After the completion of the reactions, benzylidene-based (1,3-selenazol-2-yl)hydrazones **84** were prepared in 51–91% yields (Scheme 33) [76].

The obtained selenazolyl-hydrazones **84** are selective monoamine oxidase inhibitors with antioxidant and antiproliferative activities [76].

**Scheme 34:**
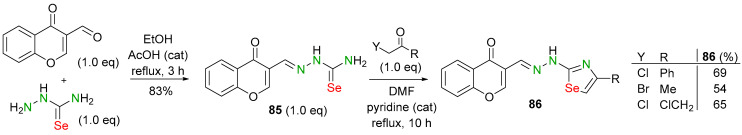
Synthesis of functionalized 1,3-selenazoles **86** with 4-oxo-4*H*-chromen-3-yl fragment.

Functionalized 1,3-selenazoles **86,** with an 4-oxo-4*H*-chromen-3-yl fragment, were constructed by the Hantzsch-type condensation reaction. This method is based on the cyclization of *N*-[(4-oxo-4*H*-chromen-3-yl)methylene]selenosemicarbazide (**85**) with bis-halogen and *α*-halocarbonyl compounds in DMF, containing a few drops of pyridine. Initial selenosemicarbazide **85** was prepared by the condensation of selenosemicarbazide and 4-oxo-4*H*-chromene-3-carboxaldehyde in absolute EtOH containing glacial acetic acid (Scheme 34) [77].

Compounds (**86)** (R = Ph, ClCH_2_) demonstrated significant cytotoxic activity against the Hep-G2, HCT-116, MCF-7, and A549 cancer cell lines [77].

**Scheme 35:**
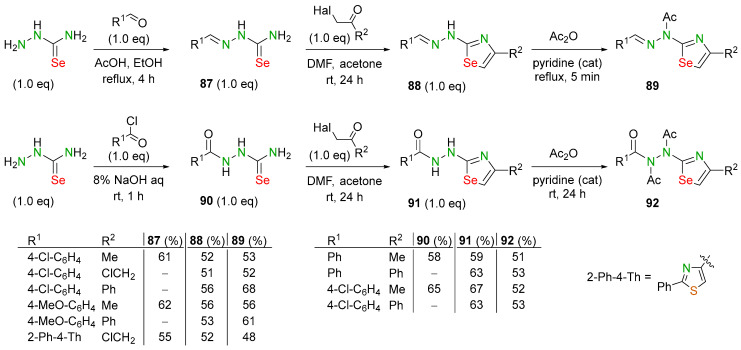
Synthesis of hydrazine functionalized 1,3-selenazoles **88**,**89**,**91**,**92**.

A series of hydrazine functionalized 1,3-selenazoles was synthesized by the Hantzsch-type condensation reaction (Scheme 35) [78]. The synthesis of the 1,3-selenazoles used two precursors, selenosemicarbazones **87** and aroyl-selenosemicarbazide derivatives **90**, prepared by the condensation of selenosemicarbazide with aromatic carbaldehydes and acylation of selenosemicarbazide, respectively.

The selenosemicarbazones **87** were further exposed to condensation using different *α*-halogenocarbonyl derivatives to yield a series of hydrazinoselenazoles **88** in 51–56% yields. The acetylation of the selenazoles **88** was readily accomplished using acetic anhydride in the presence of pyridine with the formation of selenazoles **89** in a 48–68% yield. The aroyl-hydrazinoselenazoles **91** and their diacetyl derivatives **92** were prepared in a similar way (Scheme 35) [78]. Compounds **88** and **89** (R^1^ = 2-Ph-4-Th, R^2^ = ClCH_2_) exhibited significant cytotoxic activities against two human cancer cell lines, hepatocarcinoma cells (Hep-G2) and prostate cancer cells (DU-145) [78].

**Scheme 36:**
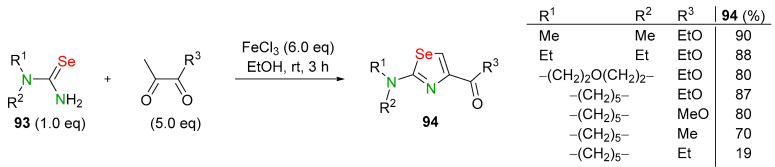
Synthesis of 2-dialkylamino-1,3-selenazoles **94**.

2-dialkylamino-1,3-selenazoles **94** were obtained in 19–90% yields by the reactions of *N*,*N*-unsubstituted selenoureas **93** with diketone derivatives in the presence of FeCl_3_. (Scheme 36) [79].

**Scheme 37:**
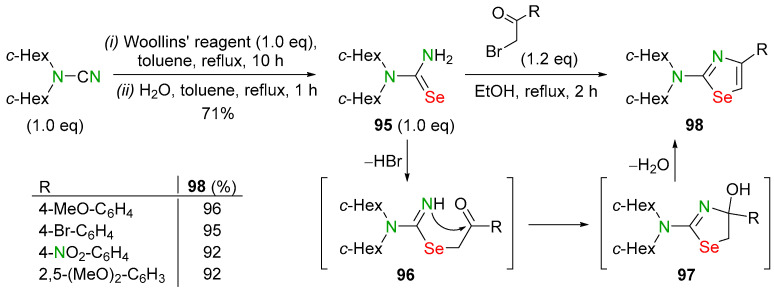
Synthesis of *N*,*N*-dicyclohexyl-4-substirured 1,3-selenazol-2-amine **98**.

The reaction of Woollins’ reagent with *N*,*N*-dicyclohexylcyanamides followed by treatment with water led to the formation of 1,1-dicyclohexylselenourea **95** (Scheme 37) [80].

Selenourea **95** was converted into *N*,*N*-dicyclohexyl-4-substituted 1,3-selenazol-2-amine **98** in excellent yields (92–96%) by subsequent cyclization with four different *α*-haloketones. The reaction pathway included the formation of the intermediates **96** and **97**. The intermediate **96**, the addition product of selenourea **95** and *α*-halohekones, was involved in the cyclization reaction resulting in another intermediate **97**. This intermediate subsequently lost one molecule of water to form compounds (**98)** (Scheme 37) [80].

**Scheme 38:**
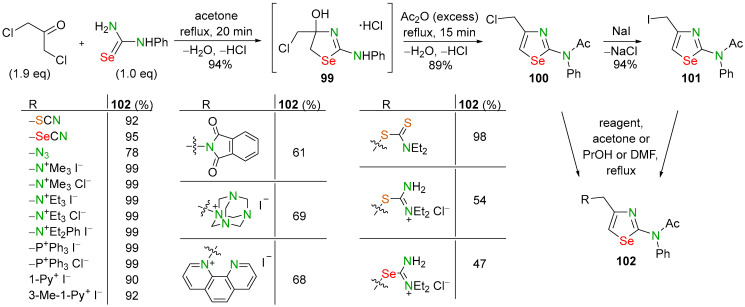
Synthesis of 4-halomethyl-2-amino-1,3-selenazoles **100**,**101** as precursors of 2,4-functionalized 1,3-selenazoles **102**.

The cyclization of *α*,*α*’-dichloroacetone with phenylselenourea afforded dihydroselenazole **99**, which was used in the efficient synthesis of selenazoles **100** and **101** (Scheme 38) [81]. Compound **99** was isolated by precipitation and used as a crude material for the preparation of selenazole **100**. The reaction of compound **99** with acetic anhydride led to the elimination of water and protection of the amino group by acetylation, yielding 4-chloromethyl-1,3-selenazole **100** in an 89% yield. The treatment of selenazole **100** with NaI afforded the highly reactive 4-iodomethyl-1,3-selenazole **101** in a 94% yield. The 4-halomethyl-2-amino-1,3-selenazoles **100** and **101** are the key intermediates for the synthesis of functionalized 1,3-selenazoles **102** (47–99% yields) (Scheme 38) [81].

**Scheme 39:**
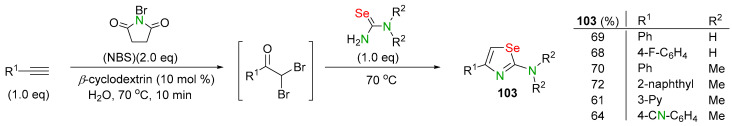
Synthesis of 4-aryl-1,3-selenazol-2-amines **103**.

The tandem one-pot synthetic protocol for the synthesis of selenazoles under mild conditions from alkynes was developed (Scheme 39) [82]. The reaction of arylacetylenes with *N*-bromosuccinimide and water led to 2,2-dibromo-1-arylethanones, which further reacted with selenoureas to afford the desired products. The reaction was catalyzed by *β*-cyclodextrin in aqueous medium and resulted in 4-aryl-1,3-selenazol-2-amines **103** (61–72% yields) (Scheme 39) [82].

It is noteworthy that water is nontoxic, economically viable, and the most readily available reaction medium. The reaction, which was carried out in water using *β*-cyclodextrin as a catalyst, met the conditions of green chemistry [82].

**Scheme 40:**
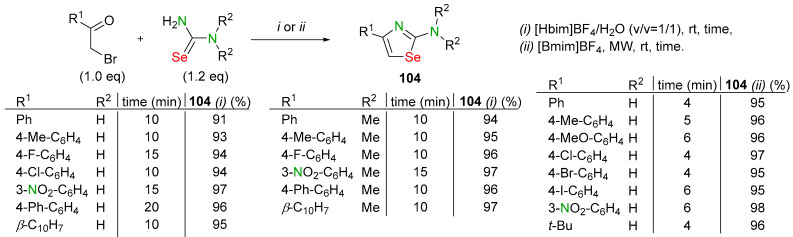
Synthesis of 2-amino-1,3-selenazoles **104** in ionic liquid/water solvent system or in ionic liquid under MW.

An efficient and environmentally tolerant synthesis of 2-amino-1,3-selenazoles **104** in an ionic liquid/water solvent system was reported (Scheme 40) [83,84]. The synthesis of 2-amino-1,3-selenazoles **104** in 91–97% yields was developed by the condensation of various phenacyl bromides with selenourea in short reaction times (4–6 min) under ambient conditions. The addition of water to ionic liquid [Hbim]BF_4_ (1-*n*-butylimidazolium tetrafluoroborate) in a 1:1 ratio enhanced the rate of cyclocondensation reaction and reduced the reaction time. This methodology has notable advantages such as the absence of any added catalyst, simple operation, and the recyclability of the ionic liquid. Moreover, acyl halides and phenacyl halides easily undergo condensation with selenourea in [Bmim]BF4 (1-butyl-3-methylimidazolium tetrafluoroborate) as ionic liquid by microwave irradiation (MW) to afford the desired products **104** in 95–98% yields (Scheme 40) [83,84].

**Scheme 41:**
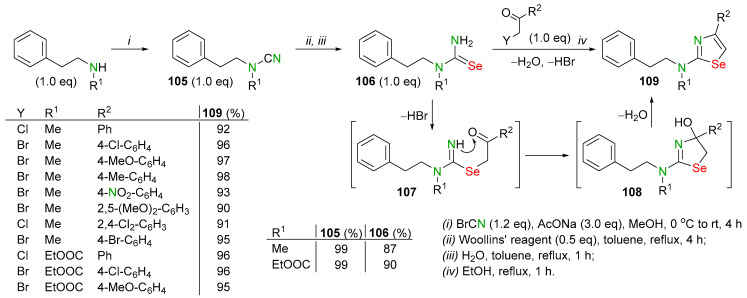
Synthesis of 4-substituted-1,3-selenazol-2-amines **109**.

A series of new 4-substituted 2-(2-phenylethyl)amino 1,3-selenazoles **109** were synthesized based on (2-phenylethyl)amines (Scheme 41) [85]. Initial cyanamides **105** were prepared in almost quantitative yields from the reaction of BrCN with secondary amines in dry MeOH in the presence of anhydrous MeCOONa at room temperature. Two selenoureas **106** were obtained in 87–90% yields by the reaction of Woollins’ reagent with cyanamides **105**, followed by post-treatment with water. The cyclization of selenoureas **106** with equimolar amounts of *α*-haloketones in refluxing ethanol solution yielded a series of 2-(2-phenylethyl)amino 1,3-selenazoles **109** in excellent yields (90–98%). The formation of products **109** occurred through intermediates **107** and **108** (Scheme 41) [85].

**Scheme 42:**
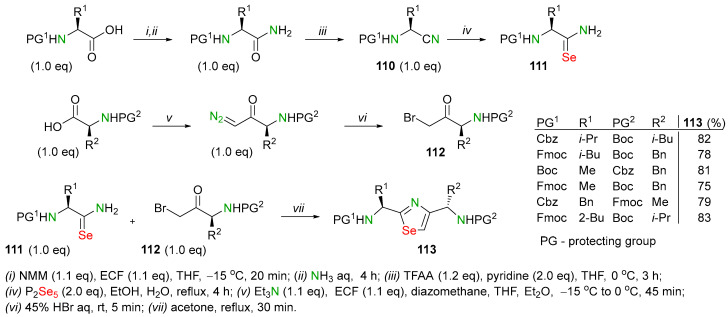
Synthesis of *N*-orthogonally protected dipeptidomimetics **113**.

A simple protocol was developed for the synthesis of *N*-orthogonally protected dipeptidomimetics **113** linked with the selenazole ring by the condensation of *N*-amino selenoamides with *α*-bromomethyl ketones (Scheme 42) [86]. The synthesis of *N*-protected amino nitriles **110** was undertaken on the first stage. *N*-Methylmorpholine (NMM) and ethyl chloroformate (ECF) were added to a solution of *N*-protected amino acids in THF and stirred at −15 °C. After the treatment with aqueous ammonia solution, amino acid amides were obtained. Next, the solution of prepared amino acid amides in dry THF was treated with trifluoroacetic anhydride (TFAA) and pyridine and the reaction mixture was stirred at 0 °C to produce nitriles **110**. The reaction of nitriles **110** with P_2_Se_5_ yielded selenoamides **111**.

Bromomethyl ketones **112** were prepared from *N*-protected amino acid, ethyl chloroformate, diazomethane and HBr (Scheme 42) [86]. *N*-orthogonally protected dipeptidomimetics **113** were obtained in 75–83% yields by the condensation of *N*-amino selenoamides **111** with *α*-bromomethyl ketones **112** in refluxing acetone.

**Scheme 43:**
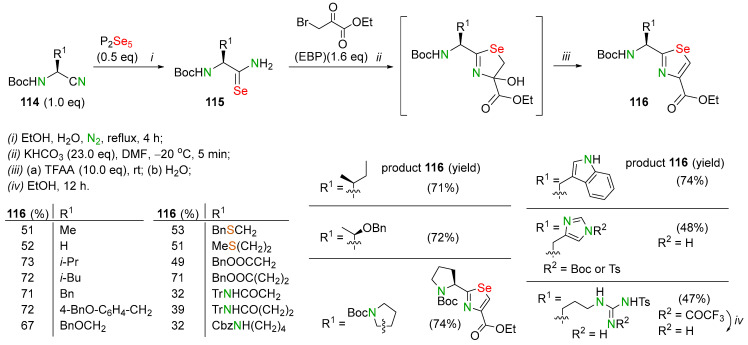
Synthesis of 2,4-substituted selenazoles **116**.

An efficient protocol for the preparation of optically pure 1,3-selenazoles has been developed (Scheme 43) [22]. Aliphatic selenoamides **115** can be prepared by refluxing a mixture of the amino-acid-derived nitriles **114** and P_2_Se_5_ in an EtOH/H_2_O mixture. However, in studies, these selenoamides **115** were unstable during purification. Therefore, after simple filtration and rapid concentration, the crude selenoamides **115** were then reacted with ethyl bromopyruvate (EBP) in the presence of excess KHCO_3_ in DMF at −20 °C for 5 min. Dehydration was followed by adding trifluoroacetic anhydride (TFAA) to the reaction mixture to obtain 2,4-substituted selenazoles **116** in 32–74% yields. These reaction conditions appeared to be important for suppressing the racemization of the final products suitable for synthesis of optically pure peptides containing 1,3-selenazole fragments (Scheme 43) [22].

**Scheme 44:**
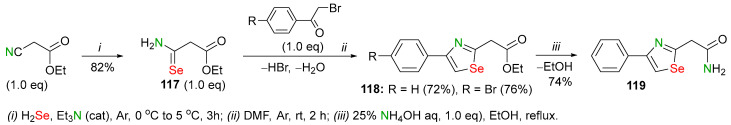
Synthesis of trisubstituted 2-amino-1,3-selenazole derivatives **119**.

A new reagent, ethyl 3-amino-3-selenoxopropanoate **117** (82% yield), was obtained from ethyl cyanoacetate and H_2_Se in the presence of Et_3_N. The reaction of product **117** with phenacyl bromides involving the selenoamide group in DMF under an argon atmosphere led to the formation of the desired 1,3-selenazoles **118**. Upon heating selenazole **118** (R = H) with ammonia in ethanol solution, the ammonolysis of the ester group occurred to afford 2-thiazole-2-acetamide **119** in a 74% yield (Scheme 44) [87].

**Scheme 45:**
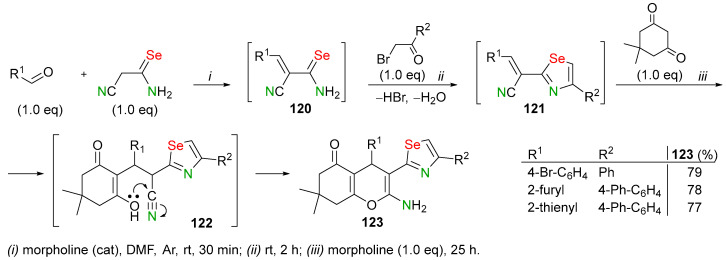
Synthesis of 2-amino-4-chromen-5(6*H*)-ones **123**.

The multicomponent condensation of aromatic aldehydes, cyanoselenoacetamide, *α*-bromoketones, and dimedone was studied (Scheme 45) [88]. The reaction was carried out in DMF in the presence of morpholine at room temperature. This cascade transformation resulted in the formation of 2-amino-4-chromen-5(6*H*)-ones **123** in 77–79% yields. The reaction involved the formation of Knoevenagel alkenes **120** as intermediates. The Hantzsch-type reaction occurred to form vinylselenazoles **121**, which further reacted with dimedone to yield intermediate **122.** The latter underwent intramolecular cyclization into the final heterocyclic system **123** (Scheme 45) [88]. 3-Aminoselenoacrylamides are promising initial materials for the preparation of selenium heterocycles.

**Scheme 46:**
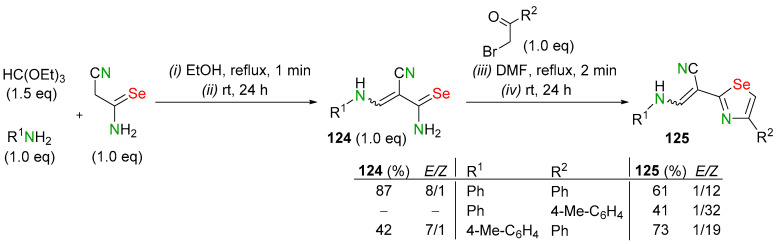
Synthesis of 2,4-substituted 1,3-selenazole derivatives **125**.

A convenient approach to producing 3-aminoselenoacrylamides **124** based on a three-component condensation reaction of triethylorthoformate, primary aromatic amines, and cyanoselenoacetamide was discovered (Scheme 46) [89].

The unsaturated selenoamides **124** were formed as a mixture of *Z*- and *E*-isomers by refluxing the reagents in EtOH. The selenoamides **124** readily took part in Hantzsch-type reactions with *α*-bromoketones to form 2,4-substituted 1,3-selenazole derivatives **125** predominantly as the *Z*-isomers (Scheme 46) [89].

**Scheme 47:**
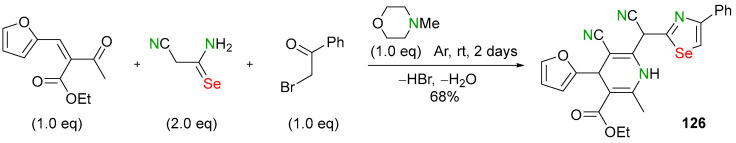
Synthesis of compound **126**.

The reactions of cyanoselenoacetamide, 2-furfurylidene acetoacetic ester, and phenacyl bromide in the presence of *N*-methylmorpholine at 20 °C under an argon atmosphere afforded functionalized selenazole **126** (68% yield), containing furan and dihydropyridine rings (Scheme 47) [90].

### 2.3. 2,5-Substituted 1,3-Selenazoles

**Scheme 48:**
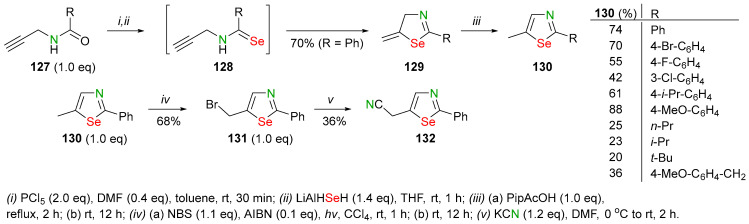
Synthesis of 2,5-disubstituted 1,3-selenazoles **130**–**132** with using Ishihara reagent.

An approach to 2,5-disubstituted 1,3-selenazoles based on the cycloisomerization of propargyl selenoamides, using readily available starting materials, was reported (Scheme 48) [91]. The intermediate selenoamides **128** were synthesized in situ from aromatic and aliphatic terminal propargyl amides **127** with neutral, electron-rich, and electron-deficient groups.

This procedure used PCl_5_ and a catalytic amount of DMF in toluene for the conversion of amides into iminochlorides, followed by the exchange of chlorine for selenium using the Ishihara reagent (LiAlHSeH), freshly prepared from elemental selenium and lithium alumohydride. The spontaneous *5-exo-dig* cyclization of intermediates **128** led to the formation of selenazolines **129**. Compound **129** (R = Ph) was isolated in 70% yield. The *exo*-double bond of compounds **129** was isomerized with piperidinium acetate, and 2,5-disubstituted 1,3-selenazoles **130** were prepared in up-to-88% yields. Methylselenazole **130** (R = Ph) was transformed into a bromomethyl derivative **131** (68% yield) using *N*-bromosuccinimide (NBS) in the presence of azobisisobutyronitrile (AIBN) in CCl_4_ with irradiation and room temperature. The cyanide **132** was obtained from intermediate **131**, which can be considered a versatile building block for further derivatizations (Scheme 48) [91].

**Scheme 49:**
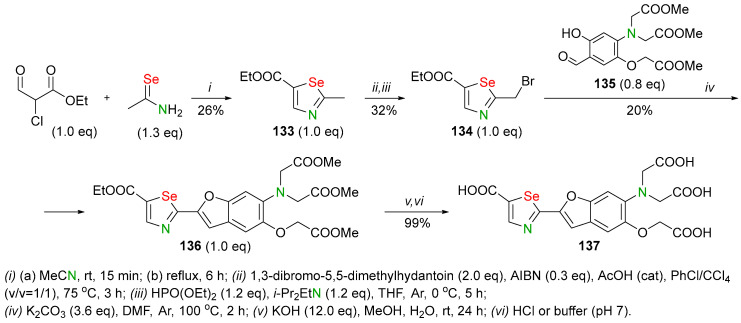
Synthesis of Se-containing ‘fura’ fluorophore **137**.

A red-shifted fluorescent indicator for magnesium was developed by the incorporation of selenium in the selenazole moiety of fura fluorophore. To introduce selenium into the structure of Mag-fura-2, 2-methyl-1,3-selenazole **133** was prepared by the reflux of freshly prepared selenoacetamide and ethyl 2-chloro-3-oxopropanoate in MeCN (Scheme 49) [92].

Following nonselective radical bromination resulted in a mixture of 2-bromomethyl- and 2-dibromomethyl-1,3-selenazoles. The treatment of the mixture with Hünig’s base and diethylphosphite made it possible to transform an undesirable dibromo by-product into pure 2-bromomethyl-1,3-selenazole **134** in a 32% yield. This precursor was used to finally produce the benzofuran fluorophore **136** (20% yield) via condensation with salicylaldehyde **135**, which contains the protected magnesium-binding group. Metal-responsive sensor **137** in the form of free acid may be generated in a quantitative yield via the base-catalyzed hydrolysis of the esters right before use. The ester form of the sensor is more stable during storage (Scheme 49) [92].

The replacement of one chalcogen atom in the acceptor oxazole fragment of the Mag-fura-2 indicator induces an increase in the Stokes shift and a large shift in emission wavelength without affecting, significantly, the basic chemical features of the compound [92].

**Scheme 50:**
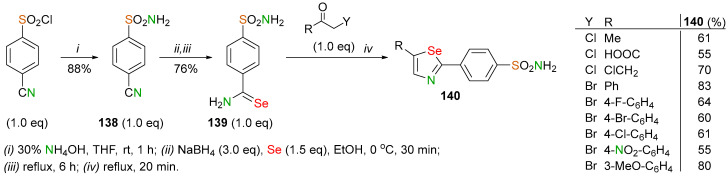
Synthesis of 2,5-disubstituted 1,3-selenazole derivatives **140** containing sulfonamide moiety.

A series of 2,5-disubstituted 1,3-selenazole derivatives, containing sulfonamide moiety, were synthesized (Scheme 50) [93,94]. 4-Cyanobenzenesulfonamide (**138**) was prepared in an 88% yield by the reaction of the corresponding sulfonyl chloride with an aqueous solution of ammonium hydroxide. Consistently, 4-sulfamoyl benzo selenoamide (**139**) was prepared in a 76% yield by the reaction of nitrile compound **138** with Na_2_Se as the selenating reagent in refluxing ethanol. And lastly, the reaction of primary selenobenzamide **139** with various *α*-haloketones, including aliphatic or aromatic moieties, yielded various 2,5-disubstituted 1,3-selenazoles **140** in 55–83% yields (Scheme 50) [93,94]. Prepared selenazoles **140** demonstrated potent inhibitory effects on cell viability against breast (MDA-MB-231) cancer cell lines and the human prostate (PC3), thus possessing effective antitumor activity. Also, compounds **140** showed antibacterial activity against *Helicobacter pylori* and *Burkholderia pseudomallei* [93,94].

**Scheme 51:**
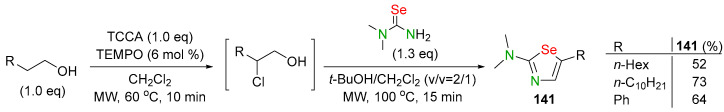
Synthesis of 5-substituted 2-amino-1,3-thiazoles **141** using MW.

Primary alcohols were converted into 5-substituted 2-amino-1,3-selenazoles **141** under microwave irradiation (MW) using trichloroisocyanuric acid (TCCA) as the chlorine source and an oxidant, (2,2,6,6-tetramethylpiperidin-1-yl)oxyl (TEMPO), as a co-oxidant, and dimethyl selenourea (Scheme 51) [95]. Primary alcohols were added to a mixture of TCCA and TEMPO in CH_2_Cl_2_ and the mixture was stirred under microwave irradiation at 60 °C for 10 min. Upon the formation of the *α*-chlorocarbonyl compound, *t*-BuOH and dimethyl selenourea were added, and the mixture was additionally stirred under microwave irradiation at 100 °C for 15 min.

**Scheme 52:**
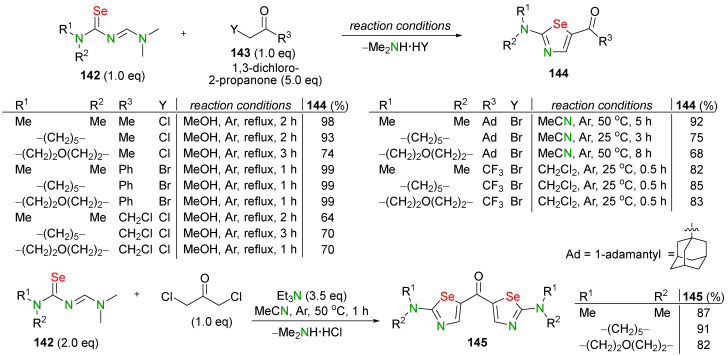
Synthesis of 5-acyl-2-amino-1,3-selenazoles **144** and bis[2-dialkylamino-5-(1,3-selenazolyl)] ketones **145**.

The reaction of selenazadienes **142** with *α*-haloketones **143** in the appropriate conditions yielded 5-acyl-2-amino-1,3-selenazoles **144** in high yields (64–99%) (Scheme 52) [96,97].

The reaction of compounds **142** with 1,3-dichloro-2-propanone in the presence of Et_3_N yielded bis [2-dialkylamino-5-(1,3-selenazolyl)] ketones **145** (82–91% yields) as major products together with 1,3-selenazoles **144** as the minor products (Scheme 52) [96,97].

According to the investigation results, bis[2-dimethylamino-5-(1,3-selenazolyl)] ketone showed the properties of effective and potentially useful superoxide anion-scavenger in vitro [97].

Five different selenazadienes (selenocarbamoylamidines) **147** were prepared in 75–98% yields by the reaction of *N*,*N*-dialkylselenoureas **146** and *N*,*N*-dimethylformamide dimethyl acetal in THF at room temperature.

The reaction of selenazadienes **147** with chloroacetyl chloride in THF yielded 1,3-selenazole-5-carboxylic acids **148** in moderate yields (40–80%). However, the expected acyl chloride derivative of 1,3-selenazole was not isolated.

**Scheme 53:**
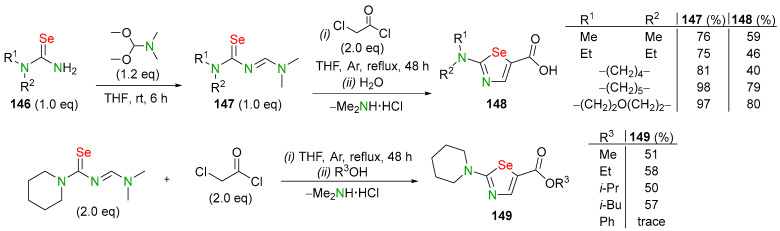
Synthesis of 1,3-selenazole-5-carboxylic acids **147** and carboxylates **149**.

The capture of the intermediate, obtained by the reaction of *N*,*N*-dimethyl-*N’*-(piperidinoselenocarbonyl)formamidine with chloroacetyl chloride, with various alcohols afforded 1,3-selenazole-5-carboxylates **149** in 50–58% yields (Scheme 53) [98,99].

**Scheme 54:**
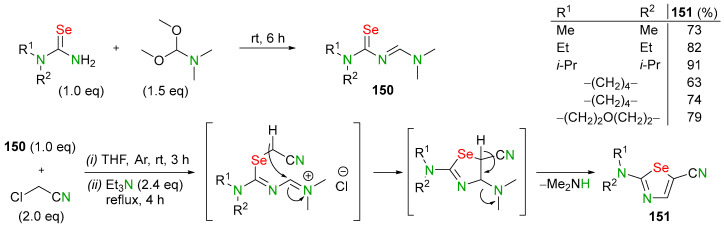
Synthesis of 2-organylamino-1,3-selenazole-5-carbonitrile **151**.

Selenazadienes **150** were prepared by the condensation of *N*,*N*-unsubstituted selenoureas with *N*,*N*-dimethylformamide dimethylacetal at room temperature. Six kinds of 1,3-selenazole-5-carbonitriles **151** were obtained by reactions of selenazadienes **150** with chloroacetonitrile in the presence of Et_3_N (Scheme 54) [100]. Chloroacetonitrile was added to a stirred solution of compound **150** in dry THF under an argon atmosphere at an ambient temperature. The reaction mixture was refluxed for 3 h. Then, Et_3_N was added and the reaction mixture was refluxed. As a result, 2-organylamino-1,3-selenazole-5-carbonitrile **151** samples were prepared in 73–91% yields.

**Scheme 55:**
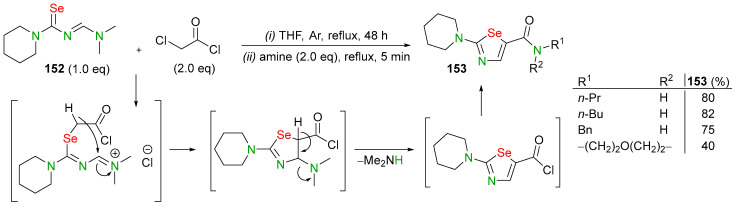
Synthesis of 1,3-selenazole-5-carboxamides **153**.

The reactions of *N*,*N*-dimethyl-*N’*-(piperidinoselenocarbonyl)-formamidine **152** with chloroacetyl chloride and then with amines yielded 1,3-selenazole-5-carboxamides **153** (Scheme 55) [100]. The reaction of chloroacetyl chloride with selenoazadienes **152** was carried out in dry THF under an argon atmosphere at room temperature. After the addition of amines, the obtained mixture was refluxed for 5 min. After the working up of the reaction mixture and purification of the residue, 1,3-selenazole-5-carboxamides **153** were prepared in 40–82% yields (Scheme 55) [100].

**Scheme 56:**
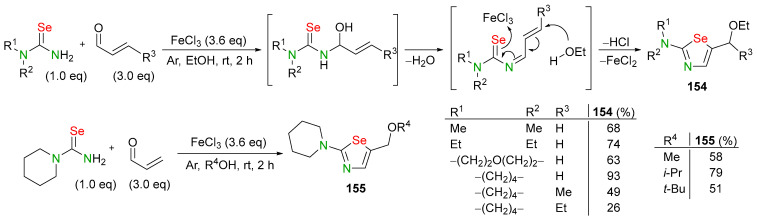
Synthesis of 2-dialkylamino-1,3-selenazoles **154**,**155** in the presence of FeCl_3_.

2-dialkylamino-1,3-selenazoles **154** were obtained in 26–93% yields by the reaction of *α*,*β*-unsaturated aldehydes with selenoureas in EtOH in the presence of FeCl_3_ at an ambient temperature (Scheme 56) [101]. The reaction of selenoureas with *α*,*β*-unsaturated aldehydes was initiated by the nucleophilic addition of the nitrogen of the selenourea to the carbonyl carbon, resulting in the formation of 2-amino-1,3-selenazoles **154**. The reactions in MeOH, *i*-PrOH, and *t*-BuOH yielded 5-(alkoxymethyl)-2-amino-1,3-selenazole derivatives **155** in 51–79% yields (Scheme 56) [101].

### 2.4. 2,4,5-Substituted 1,3-Selenazoles

**Scheme 57:**
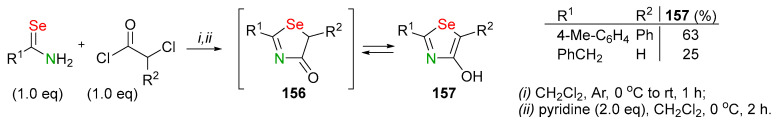
Synthesis of 2,5-substituted 4-hydroxy-1,3-selenazoles **157**.

The reaction of primary selenoamides with chloroacetyl chlorides afforded two products, 4-hydroxy-1,3-selenazoles **157,** which constitute the tautomeric enol form of compounds (**156**) (Scheme 57) [102].

**Scheme 58:**
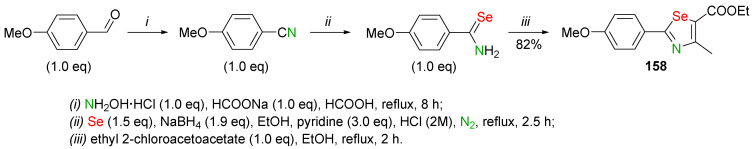
Synthesis of 2-(4-methoxyphenyl)-4-methyl-1,3-selenazole-5-ethyl carbonate **158**.

2-(4-Methoxyphenyl)-4-methyl-1,3-selenazole-5-ethyl carbonate **158** was synthesized starting from 4-methoxybenzaldehyde via intermediate 4-methoxybenzonitrile and 4-methoxybenzenecarboselenoamide (Scheme 58) [103,104]. 1,3-selenazole-5-ethyl carbonate **158** was obtained in an 82% yield by heating the mixture of 4-methoxybenzenecarboselenoamide and ethyl 2-chloroacetoacetate in EtOH under reflux. Selenazole **158** was used as a template for the synthesis of various 2,4,5-subtituted selenazoles modified by oxadiazole, pyrazole,1,2,4-triazole, tetrazole, 1,2,4-triazine, and succinic imide (Scheme 58) [103,104].

Some of the modified 1,3-selenazoles have shown the inhibitory activities against cell division cycle 25B phosphatase (Cdc25B) and can be anticancer drugs leading compounds [103].

**Scheme 59:**
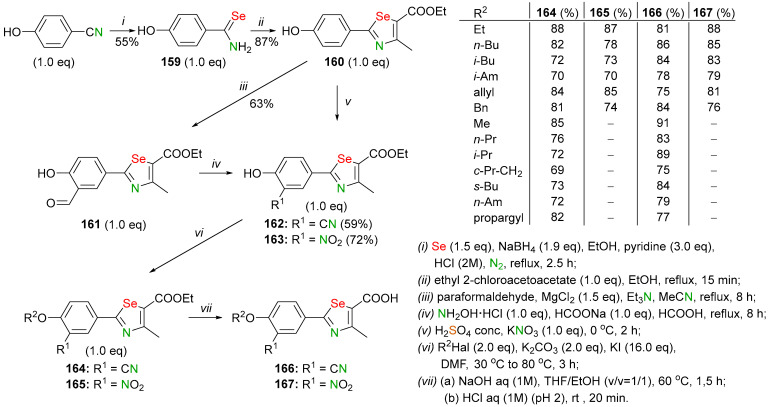
Synthesis of 2-phenyl-4-methyl-1,3-selenazole-5-carboxylic acid derivatives **166**,**167**.

A number of new 2-phenyl-4-methyl-1,3-selenazole-5-carboxylic acid derivatives **166** and **167** were synthesized (Scheme 59) [105]. Commercially accessible 4-hydroxybenzonitrile as the starting material was transformed into selenocarboxamide **159** (55% yield) by reaction with NaHSe. The freshly prepared product **159** underwent cyclization with ethyl 2-chloroacetoacetate in refluxing ethanol to form the key intermediate **160** in 87% yield. The ortho-formylation of compound **160** with paraformaldehyde in the presence of anhydrous MgCl_2_ and Et_3_N was used for the synthesis of salicylaldehyde **161** with a high selectivity and isolated yield (63%). The reaction of salicylaldehyde **161** with NH_2_OH∙HCl using sodium formate as a base under reflux in formic acid provided compound **162** in a 59% yield. On the other hand, product **163** (72% yield) was prepared by the treatment of intermediate **160** with KNO_3_ in concentrated H_2_SO_4_. Compounds **162** and **163** were alkylated with corresponding alkyl halide in DMF to provide 1,3-selenazoles **164** and **165**, which were subsequently hydrolyzed in the presence of NaOH followed by acidification to obtain the target compounds **166** and **167** (Scheme 59) [105].

Most of the products exhibited inhibitory activity against xanthine oxidase in vitro in the nanomolar range. Compound **166** (R = allyl) is considered the most potent xanthine oxidase inhibitor [105].

**Scheme 60:**
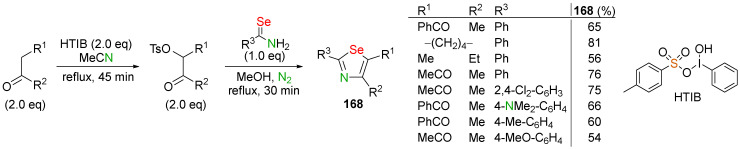
Synthesis of 2,4,5-Substituted 1,3-selenazoles **168** with using HTIB.

*α*-Tosyloxylketones, prepared by the treatment of ketones with HTIB, were involved in a reaction with primary selenoamides to provide a convenient method of synthesis of 1,3-selenazoles (Scheme 60) [52]. This method avoids the use of toxic and lachrymatory *α*-haloketones. 2,4,5-Substituted 1,3-selenazoles **168** were prepared in 54–81% yields from the corresponding ketones and selenoamides under reflux in a solvent mixture MeCN/MeOH.

**Scheme 61:**
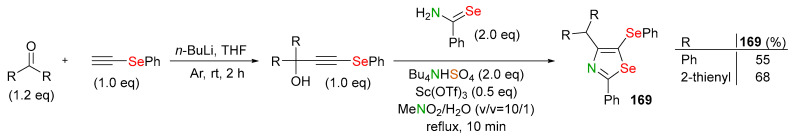
Scandium-catalyzed synthesis of 4-bisarylmethyl-1,3-thiazoles **169**.

Selenazole syntheses by the Sc-catalyzed cycloaddition reactions of 1,1-bisaryl-3-phenylselanylpropargyl alcohols with selenoamides was reported (Scheme 61) [106]. The reaction of selenamide with 1,1-bisarylpropargyl alcohols in MeNO_2_/H_2_O resulted in 4-bisarylmethyl-1,3-selenazoles **169**. Initial 1,1-bisaryl-3-(phenylselanyl)prop-2-yn-1-ols were obtained using *n*-BuLi by the reaction of ethynyl phenyl selenide with diarylketones in THF under an argon atmosphere at room temperature (Scheme 61) [106].

**Scheme 62:**
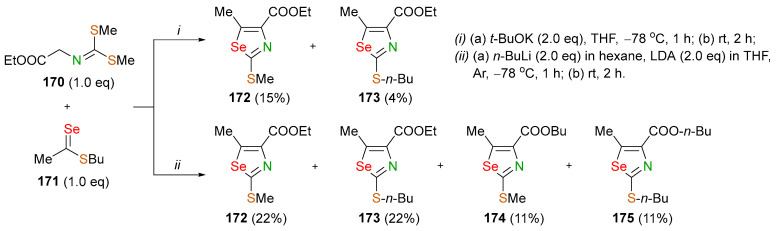
Synthesis of 2-alkylthio-4,5-disubstituted-1,3-selenazoles **172**–**175**.

The synthesis of 2-alkylthio-4,5-disubstituted-1,3-selenazoles **172**–**175** via the metallation of dimethyl *N*-(ethoxycarbonylmethyl)iminodithiocarbonate and further reaction with selenothioic acid *S*-ester was developed (Scheme 62) [107]. Using the LDA–THF system for the deprotonation of compound **170**, selenazoles **172** and **173** in 22% yields were isolated after chromatography on silica gel. In addition, two products, **174** and **175,** were isolated. This reaction afforded four 2-alkylthio-substituted selenazoles **172**–**175**, which varied only by side chains attaching to the C-2 and C-4 functional groups (Scheme 62) [107].

These selenazoles **172**–**175** are easily transformed into electron-rich diselenadiazafulvalenes and azino-diselenadiazafulvalenes with excellent reversible donor ability [107].

**Scheme 63:**
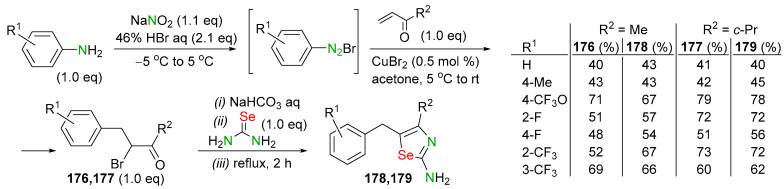
Synthesis of 2-amino-4-alkyl-5-(*R*-benzyl)selenazoles **178**,**179**.

A one-pot synthesis of 2-amino-4-alkyl-5-(*R*-benzyl)selenazoles **178** and **179** was developed through the bromoarylation of methyl or cyclopropyl vinyl ketones and further cyclization with selenourea (Scheme 63) [108]. Anilines were first converted into arenediazonium bromides, which reacted with methyl vinyl ketone or cyclopropyl vinyl ketone in the presence of CuBr_2_ to produce 4-aryl-3-bromobutan-2-ones **176** (40–71% yields) and 3-aryl-2-bromo-1-cyclopropylpropan-1-ones **177** (41–79% yields). These products reacted with selenourea without preliminary isolation. After nitrogen was no longer evolved, the reaction mixture was neutralized with NaHCO_3_ and selenourea was added to the reaction mixture, which was heated at reflux temperature for 2 h. The 4-methyl-5-(*R*-benzyl)selenazol-2-amines **178** and 4-cyclopropyl-5-(*R*-benzyl)selenazol-2-amines **179** were prepared in 43–67% and 40–72% yields, respectively (Scheme 63) [108].

**Scheme 64:**
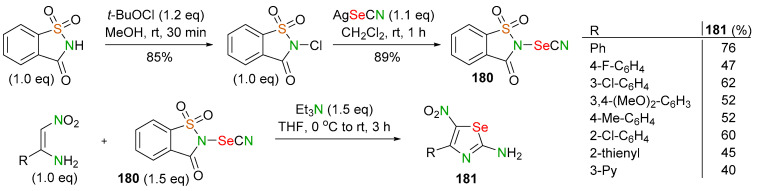
Synthesis of 4-aryl-substituted 2-amino-5-nitro-1,3-selenazoles **181**.

An efficient and convenient protocol for the direct synthesis of 4-substituted 2-amino-5-nitro-1,3-selenazoles **181** from *β*-aminonitroalkenes and *N*-selenocyanatosaccharin **180** was developed (Scheme 64) [109]. *N*-selenocyanatosaccharin reagent was prepared from saccharin by the treatment with *tert*-butyl hypochlorite in MeOH and then with AgSeCN in CH_2_Cl_2_ at room temperature. Freshly prepared selenocyanatosaccharin **180** was added to *β*-nitroenamine in anhydrous THF at 0 °C and the reaction mixture was stirred for 30 min. Then, triethylamine was added, and the reaction mixture was stirred at room temperature for 3 h. After workup, 4-aryl-substituted 2-amino-5-nitro-1,3-selenazoles **181** were isolated in 40–76% yields. This metal-free method is convenient and features a short reaction time, mild reaction conditions, and a good compatibility of functional groups (Scheme 64) [109].

**Scheme 65:**
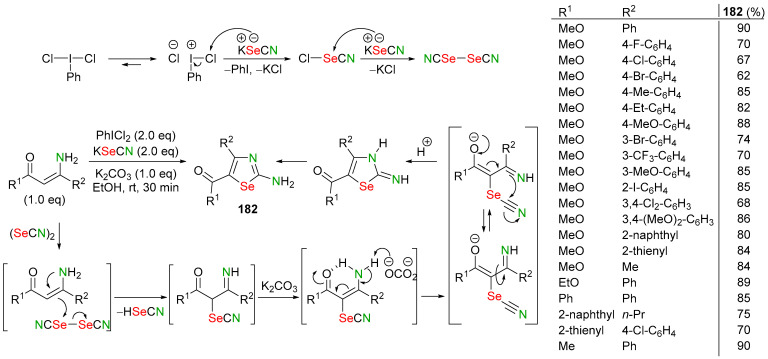
Synthesis of 4,5-substituted 2-amino-1,3-selenazoles **182** via selenocyanative cyclization of enamino esters and enaminones.

An alternative one-pot method for the synthesis of the biologically promising 2-amino-1,3-selenazole skeleton under mild conditions was demonstrated (Scheme 65) [110]. The formation of a 2-amino-1,3-selenazole skeleton was made by the electrophilic selenocyanation of *β*-enaminones and *β*-enamino esters followed by intramolecular cyclization under basic conditions. The electrophilic compound, selenocyanogen, was obtained in situ from the reaction of PhICl_2_ and KSeCN. Thus, 4,5-substituted 2-amino-1,3-selenazoles **182** were prepared in 62–90% yields. This method is based on the use of the easily accessible selenocynate salt as both the selenium and nitrogen sources for the formation of the 1,3-selenazole ring (Scheme 65) [110]. On the other hand, the classical Hantzsch approach involves the use of selenourea or selenoamide as the building block.

**Scheme 66:**
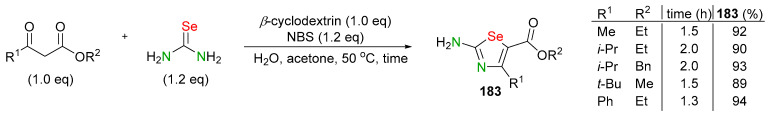
Synthesis of 2-amino-4-alkyl- and 2-amino-4-arylselenazole-5-carboxylates **183** in the presence of *β*-cyclodextrin.

Cyclodextrins selectively catalyze reactions involving supramolecular catalysis. These reactions can effectively take place in water without the formation of any toxic wastes. In this way, 2-amino-4-alkyl- and 2-amino-4-arylselenazole-5-carboxylates **183** were synthesized in studies. The reactions were carried out by the formation of the *β*-cyclodextrin complex of the *β*-ketoester in water in situ, followed by the addition of NBS (*N*-bromosuccinimide) and selenourea. Stirring at the same temperature yielded selenazole derivatives **183** in 89–94% yields. Succinimide was obtained as a by-product, and this could be recycled to produce NBS, and *β*-cyclodextrin could be easily recovered and reused (Scheme 66) [111].

**Scheme 67:**
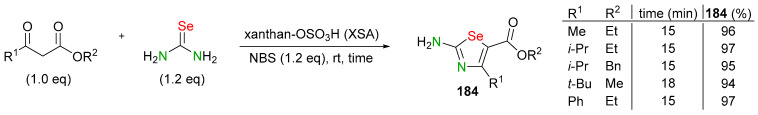
Synthesis of selenazole-5-carboxylates **184** by using xanthan sulfuric acid.

An efficient protocol has been developed for the synthesis of selenazole-5-carboxylates **184** (94–96% yields) from *β*-ketoesters, selenourea, and *N*-bromosuccinamide (NBS) by using xanthan sulfuric acid (XSA) as an effective solid acid catalyst. The reaction process is very simple and the catalyst can be easily isolated from the reaction mixture and reused several times (Scheme 67) [112].

**Scheme 68:**
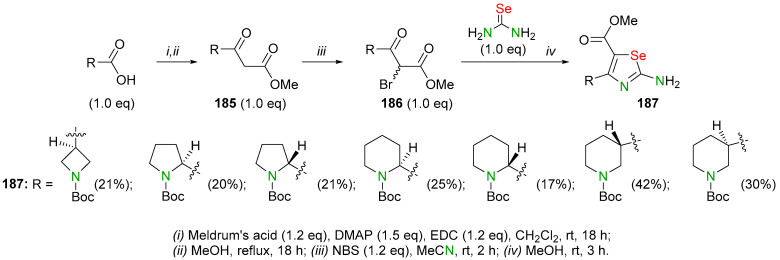
Synthesis of 2-amino-1,3-selenazole-5-carboxylates **187**.

A series of methyl 2-amino-1,3-selenazole-5-carboxylates **187** bearing chiral azetidine-3-yl, pyrrolidin-2-yl, piperidin-2-yl, or piperidin-3-yl substituents at the C4 atom of the 1,3-selenazole ring were designed and synthesized (Scheme 68). The treatment of the carboxylic chiral heterocyclic amino acid with Meldrum’s acid and 1-ethyl-3-(3-dimethylaminopropyl)carbodiimide (EDC) in the presence of 4-dimethylaminopyridine (DMAP), followed by heating the intermediate isolated from the reaction mixture in MeOH, afforded *β*-ketoesters **185**, which were then *α*-brominated with *N*-bromosuccinimide (NBS) in MeCN. The reaction of compounds **186** with selenourea at room temperature in MeOH yielded, to the target, methyl 2-amino-1,3-selenazole-5-carboxylates **187** in 17–42% yields (Scheme 68) [113,114].

Prepared chiral amino acids **187** can probably be used as starting materials for the synthesis of more complex molecules with biological activity or be used as building blocks for the development of DNA-encoded chemical libraries [113,114].

**Scheme 69:**
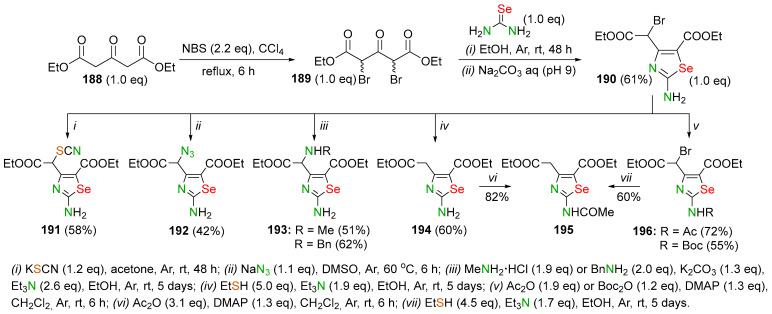
Synthesis of ethyl 2-(2-amino-5-ethoxycarbonyl-1,3-selenazol-4-yl)-2-bromoethanoate (**190**) and its derivatives **191**–**196**.

The reaction of diethyl 2,4-dibromo-3-oxoglutarate **189** with selenourea afforded ethyl 2-(2-amino-5-ethoxycarbonyl-1,3-selenazol-4-yl)-2-bromoethanoate (**190**) (Scheme 69) [115]. At first, the dibromination of diethyl 3-oxoglutarate **188** with NBS in CCl_4_ produced diethyl 2,4-dibromo-3-oxoglutarate **189** as a mixture of diastereomers, which were immediately used in the next reaction stage. The reaction of the dibrominated carbonyl compound **189** with selenourea was carried out under an argon atmosphere in EtOH at room temperature. After the working up of the reaction mixture and purifying the crude product, ethyl 2-(2-amino-5-ethoxycarbonyl-1,3-selenazol-4-yl)-2-bromoethanoate (**190**) was prepared in a 61% yield. Selenazole **190** is a convenient starting material for the synthesis of other 4,5-functionalized 2-amino-1,3-selenazoles. The treatment of selenazole **190** with acylating agents and various nucleophiles made it possible to obtain a series of new 4,5-disubstituted 2-amino-1,3-selenazoles **191**–**196** in 42–72% yields (Scheme 69) [115].

**Scheme 70:**
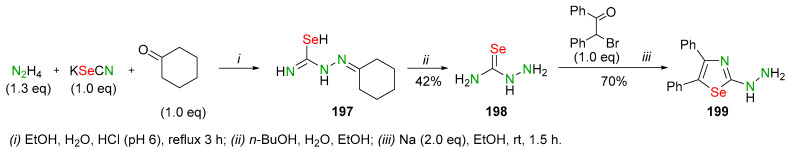
Synthesis of 2-hydrazino-4,5-diphenyl-1,3-selenazole (**199**).

The cyclization of selenosemicarbazide (**198**) with desyl bromide resulted in 2-hydrazino-4,5-diphenyl-1,3-selenazole (**199**) in a 70% yield. Selenosemicarbazide (**198**) was obtained in a 42% yield through the intermediate cyclohexanone selenosemicarbazone (**197**) by the reflux of hydrazine hydrate with potassium selenocyanate and cyclohexanone in EtOH and further treatment of the compound **197** with *n*-BuOH, water, and EtOH (Scheme 70) [50,116]. Compound **199** is a useful building block for the synthesis of functionalized selenazoles such as 1,3-selenazoles substituted with pyrazole [50].

**Scheme 71:**
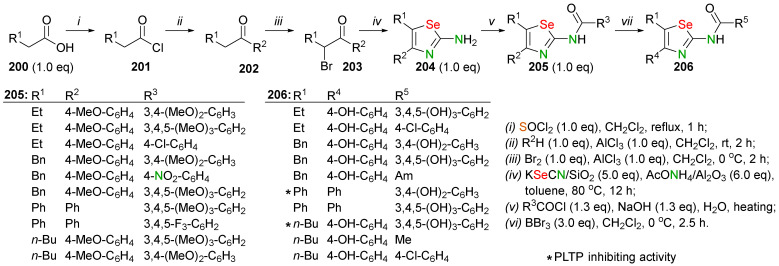
Synthesis of 2,4,5-trisubstituted-1,3-selenazoles **205** and **206**.

A series of 2,4,5-trisubstituted selenazole compounds were designed and synthesized (Scheme 71) [117,118,119]. The starting carboxylic acids **200** were treated with SOCl_2_ to yield acetyl chlorides **201**, which, without further purification, were reacted with aromatic compounds to yield products (**202)**. Then, ketones **202** were treated with bromine to yield compounds (**203)**. A suspension of products (**203)**, KSeCN/SiO_2_, and AcONH_4_/Al_2_O_3_ was heated in toluene at 80 °C to afford 2,4,5-trisubstituted-1,3-selenazoles **204** with the amino group. Then, compounds (**204)** were acylated to yield 2,4,5-trisubstituted 1,3-selenazoles **205**. If compounds (**205)** had the methoxyl group, these compounds were treated with BBr_3_ to provide phenolic compounds (**206)**. Unfortunately, the yields of compounds **205** and **206** were not indicated (Scheme 71) [117,118,119].

Some examples of compounds (**206)** exhibited favorable PLTP (plasma phospholipid transfer protein)-inhibiting activity [119].

**Scheme 72:**
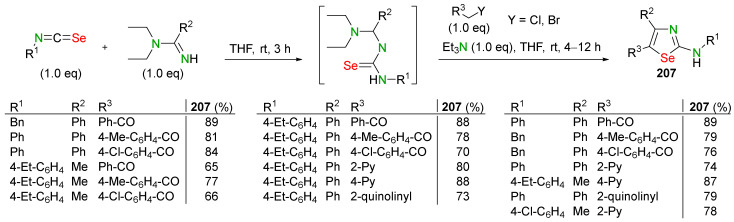
Synthesis of trisubstituted 2-amino-1,3-selenazole derivatives **207**.

A consistent multicomponent one-pot reaction for the synthesis of 2-amino-1,3-selenazole **207** was developed (Scheme 72) [120]. *N,N*-Diethyl-benzamidine or acetamidine was added to a solution of isoselenocyanate in THF, and the resulting reaction mixture was stirred for 3 h at room temperature. To this reaction mixture, Et_3_N was added, followed by the addition of halomethylene compound in THF, and the stirring continued until the completion of the reaction. Thus, amidino-selenourea synthesized from isoselenocyanate and *N,N*-diethyl-amidine reacted with various halomethylene compounds to furnish 2-amino-1,3-selenazole derivatives **207** in 65–89% yields (Scheme 72) [120].

**Scheme 73:**
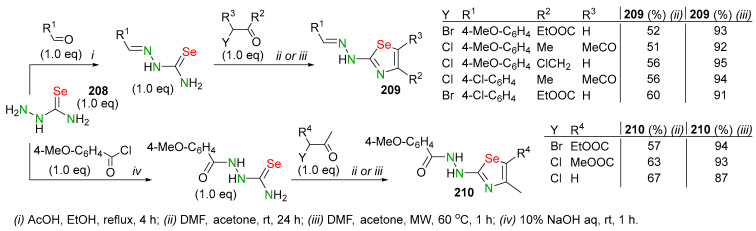
Synthesis of aryl-hydrazinyl-1,3-selenazoles **209** and aroyl-hydrazonyl-1,3-selenazoles **210**.

Aryl-hydrazinyl-1,3-selenazoles **209** and aroyl-hydrazonyl-1,3-selenazoles **210** were obtained through the Hantzsch-type condensation reactions of *α*-halogenocarbonyl derivatives with selenosemicarbazides **208** under ordinary and microwave (MW) heating conditions (Scheme 73) [121]. Shorter reaction times and excellent yields were achieved under irradiation conditions. Starting arylidenselenosemicarbazones and 4-methoxybenzoylselenosemicarbazide were obtained by the reaction of methoxybenzoyl chloride or aromatic carbaldehydes with selenosemicarbazide.

Selenazoles **209**, **210** showed moderate antiproliferative in vitro activity against two leukemia cell lines (HL60 and CCRF-CEM) and three carcinoma cell lines (HCT116, MDA-MB231, and U87MG) [121].

**Scheme 74:**
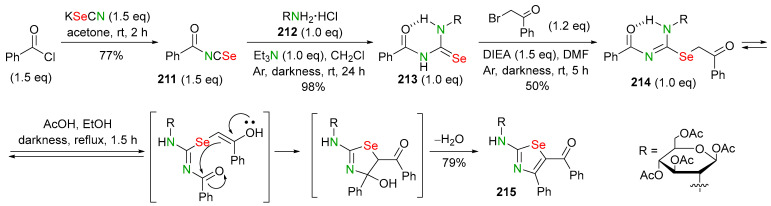
Synthesis of sugar derivatives of 2-amino-1,3-selenazole **215**.

Sugar derivatives of 2-amino-1,3-selenazole **215** were prepared as promising biologically active compounds (Scheme 74) [122]. The key intermediate for accessing such scaffold was *N*-benzoyl isoselenocyanate **211**, easily available by the treatment of potassium selenocyanate with benzoyl chloride. The coupling of compound **211** with hydrochloride **212** in CH_2_Cl_2_ in the presence of Et_3_N afforded *N*-benzoyl selenourea **213** in a 98% yield. The treatment of selenourea **213** with phenacyl bromide in DMF at room temperature in the presence of diisopropylethylamine (DIEA) afforded phenacyl isoselenourea **214** in a 50% yield. The acidic treatment of isoselenourea **214**, using refluxing ethanolic AcOH, led to an intramolecular cyclodehydration to produce selenazole **215** in a 79% yield. The construction mechanism of compound **215** can be explained by the formation of appropriate intermediates (Scheme 74) [122].

**Scheme 75:**
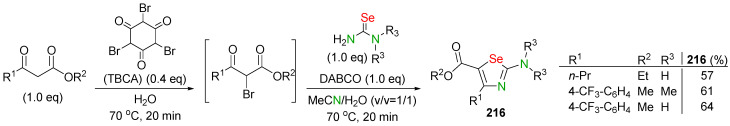
Synthesis of 4,5-substituted 2-aminoselenazoles **216** using TBCA and DABCO.

A convenient one-pot protocol for the synthesis of 2-amino selenazoles was developed that avoided using hazardous *α*-bromocarbonyl compounds (Scheme 75) [123]. The role of tribromoisocyanuric acid (TBCA) in the reaction was to produce *α*-bromo-*β*-ketoester in situ, which, in the presence of various selenoureas and 1,4-diazabicyclo[2.2.2]octane (DABCO), yielded the corresponding selenazoles. TBCA was added to a solution of *β*-ketoester in H_2_O and the mixture was stirred at 70 °C for 20 min. Then, MeCN, the selenourea, and DABCO were added, and the mixture was stirred at 70 °C for 20 min. After the working up of the reaction mixture, three 4,5-substituted 2-aminoselenazoles **216** were prepared in 57–64% yields. Short reaction times, experimental simplicity, and easily accessible reagents make this protocol attractive for the synthesis of 1,3-selenazole-containing biologically active compounds (Scheme 75) [123].

**Scheme 76:**
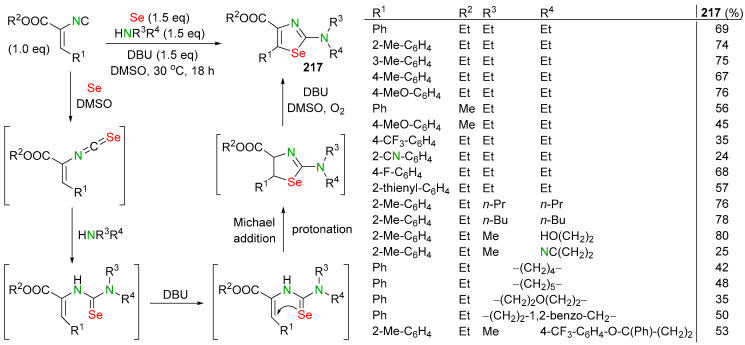
Synthesis of 2-amino-1,3-selenazole derivatives **217**.

A series of 2-amino-1,3-selenazole derivatives were synthesized using a transition-metal-free, multicomponent reaction through a base-promoted cascade cyclization process (Scheme 76) [124]. 2-amino-1,3-selenazoles **217** were obtained in 24–80% yields by the nucleophilic addition of amines to isoselenocyanate obtained in situ from elemental selenium and *α*,*β*-unsaturated isocyanides, followed by the Michael addition reaction and aromatization.

**Scheme 77:**
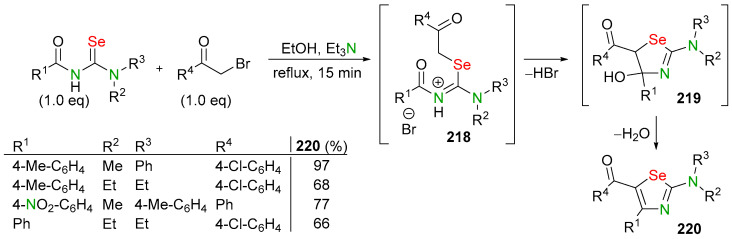
Synthesis of 5-acyl-1,3-selenazol-2-amines **220**.

5-acyl-1,3-selenazol-2-amines **220** were prepared from the reaction of 3,3-disubstituted 1-acylselenoureas and phenacyl bromides in the presence of Et_3_N in refluxing ethanol for 15 min in 66–97% yields. At first, the reaction of acyl selenoureas with phenacyl bromide yielded salts **218**. The further Hantzsch-type cyclization of salt **218** was accompanied by the formation of intermediate heterocycle **219** with the elimination of HBr. The hydration of heterocycle **219** led to the formation of 1,3-selenazoles **220** (Scheme 77) [125].

**Scheme 78:**
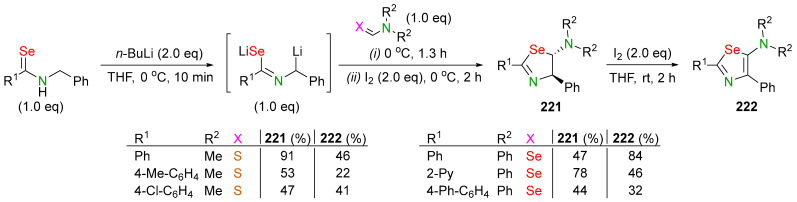
Synthesis of *N*,*N*-disubstituted 5-amino-1,3-selenazoles **222**.

The synthetic methodology for the preparation of *N*,*N*-disubstituted 5-amino-1,3-selenazoles **222** was developed. The addition reaction of selenoamide dianions to thio- and selenoformamide in THF followed by treatment with iodine at 0 °C allowed researchers to produce 5-amino-2-selenazolines **221** in 44–91% yields. Further oxidation with iodine at room temperature afforded the 5-amino selenazoles **222** in 22–84% yields (Scheme 78) [126].

**Scheme 79:**
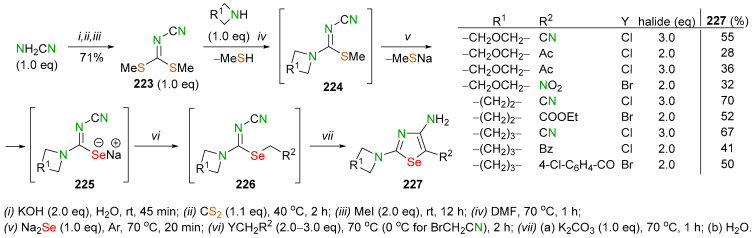
Synthesis of 2,4,5-trisubstituted-1,3-selenazoles **227**.

A very convenient procedure for the synthesis of 2,4,5-trisubstituted-1,3-selenazoles **227** was described (Scheme 79) [127,128]. The starting material for the preparation of selenazoles **227,** dimethyl cyanothioimidocarbonate **223,** was obtained in a good yield (71%) from cyanamide. Compound **223** was heated at 70 °C with secondary amines to form intermediates **224**. Freshly prepared Na_2_Se was added to the reaction mixture under an argon atmosphere at 70 °C to form the intermediate selenolates **225**. The cyclization of the intermediate **226** was carried out in the presence of K_2_CO_3_. The compounds **227** were isolated as solids in 48–87% yields (Scheme 79) [127,128].

**Scheme 80:**
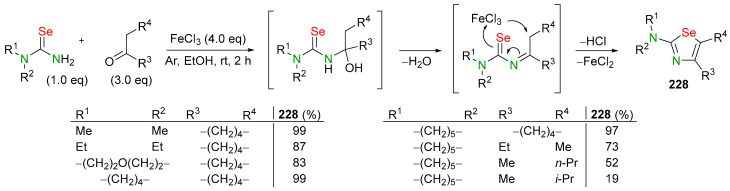
Synthesis of 4,5-substituted 2-amino-1,3-selenazoles **228** in the presence of FeCl_3_.

2-dialkylamino-1,3-selenazoles **228** were produced by the reaction of ketones with *N*,*N*-unsubstituted selenoureas in the presence of FeCl_3_. The reaction of ketones with *N*,*N*-unsubstituted selenoureas is activated by the nucleophilic addition of the nitrogen atom of the selenourea to the carbonyl carbon, resulting in the formation of 4,5-substituted 2-amino-1,3-selenazoles **228**. Selenazoles **228** were indicated to be potentially useful superoxide anion scavengers (Scheme 80) [129,130].

**Scheme 81:**
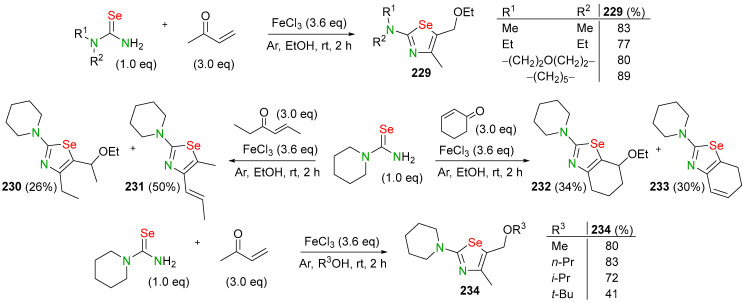
Synthesis of 4,5-substituted 2-amino-1,3-selenazoles **229–234** from *α*,*β*-unsaturated ketones in the presence of FeCl_3_.

The reaction of selenoureas with methyl vinyl ketone yielded 2-amino-5-(1-ethoxymethyl)-1,3-selenazoles **229** (77–89% yields) as the only products (Scheme 81) [131]. The reaction of piperidine-1-carboselenoamide with (2*E*)-4-methylhex-2-ene yielded two types of selenazole derivatives, 2-piperidino-5-(1-ethoxyalkyl)-1,3-selenazoles **230** and 2-piperidino-1,3-selenazoles **231**, in moderate yields. The reaction with cyclohex-2-en-1-one proceeded in a similar manner to form products **232** and **233**.

Thus, selenoureas reacted with *α*,*β*-unsaturated ketones at both *α*-positions of carbonyl carbon to yield two types of products in a certain ratio. The reactions of piperidine-1-carboselenoamide with methyl vinyl ketone in MeOH, *n*-PrOH, *i*-PrOH, and *t*-BuOH led to the formation of 2-amino-5-(1-alkoxymethyl)-1,3-selenazole derivatives **234** in 41–83% yields (Scheme 81) [131].

**Scheme 82:**
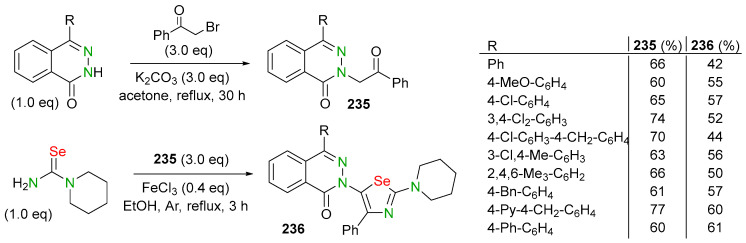
Synthesis of 1,3-selenazolyl phthalazinone derivatives **236**.

A series of 1,3-selenazoles substituted with phthalazinone derivatives were prepared (Scheme 82) [132]. The treatment of phthalazin-1(2*H*)-one derivative with phenacyl bromide in refluxing acetone in the presence of K_2_CO_3_ afforded the ketones **235** in 60–77% yields.

The reaction of 4-substituted 2-(2-phenylethyl)phthalazin-1(2*H*)-ones **235** with piperidine-containing selenoamide in EtOH under an argon atmosphere in the presence of FeCl_3_ afforded 1,3-selenazolyl phthalazinone derivatives **236** in 42–61% yields (Scheme 82) [132].

An antimicrobial activity study showed that all synthesized 1,3-selenazoles **236** exhibited moderate-to-good antifungal and antibacterial activities against pathogenic strains [132].

**Scheme 83:**
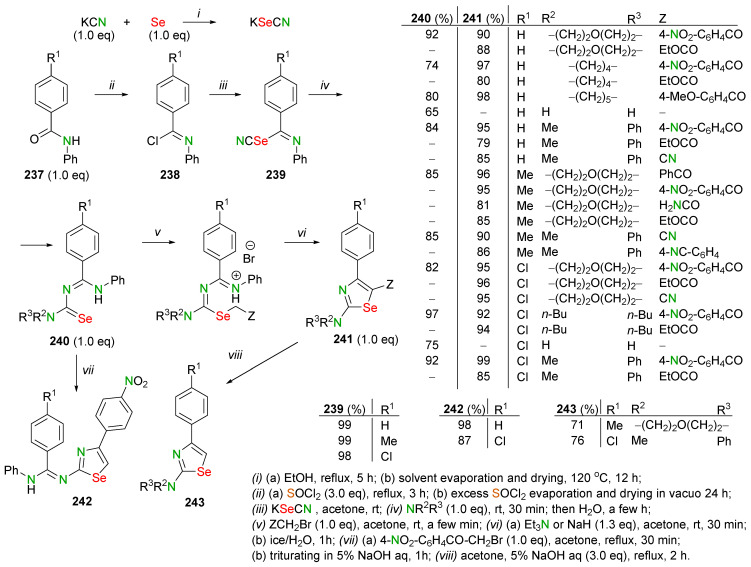
Synthesis of 1,3-selenazoles **237–243**.

4-aryl-1,3-selenazol-2-amines **241** with electron-withdrawing groups are conveniently accessible via the reaction of selenourea derivatives **240** with activated bromomethylene compounds (Scheme 83) [133].

The reaction of *N*-phenylbenzamides **237** with excess SOCl_2_ under reflux yielded *N*-phenylbenzimidoyl chlorides **238**, which, upon treatment with KSeCN in acetone, produced imidoyl isoselenocyanates **239** in quantitative yields. They were converted into selenourea derivatives **240** by reaction with primary and secondary amines or NH_3_. Compounds (**240)** reacted with activated bromomethylene compounds such as acetamides, acetonitriles, and 2-bromoacetates, as well as 4-cyanobenzyl bromide and phenacyl bromides, to yield 1,3-selenazol-2-amines **241** in 80–99% yields. The described synthesis of 1,3-selenazoles **241** is cheap and avoids using toxic compounds.

The reaction of selenourea derivatives **240** with an unsubstituted NH_2_ group led to 1,3-selenazoles **242** in up to 98% yields. In this case, an alternative ring closure process occurred. On treatment with 5% aqueous NaOH solution, ethyl 1,3-selenazole-5-carboxylates **241** were saponified and decarboxylated to yield 5-unsubstituted 1,3-selenazoles **243** (Scheme 83) [133].

## 3. Conclusions

The analysis of literature data shows that the chemistry of 1,3-selenazoles, including the study of their properties and especially biological activity, is of increasing interest to scientists.

There are examples of reactions using catalytic systems with FeCl_3_, CuBr_2_, Sc(OTf)_3_, PEG-400, benzyl triethylammonium chloride (BTEAC), NaF, [Ir(cod)Cl]_2_, *β*-cyclodextrin, PCl_5_, and xanthan sulfuric acid (XSA). The use of ultrasound or microwave irradiation significantly reduces the reaction time and increases the yields of the target products. The Hantzsch reaction has continued its new development including various modifications in the synthesis of 1,3-selenazoles.

A large number of Se-containing compounds are used as selenation agents, including elemental Se, NaHSe (usually obtained from Se and NaBH_4_), KSeCN, and Woollins’ reagent.

Most of the developed methods relate to 2,4-substituted 1,3-selenazoles. Many reactions directed to 2,4-substituted 1,3-selenazole derivatives have used *α*-bromoketones, *α*-bromaldehydes, selenoureas, and selenoamides as starting reagents. Regarding the starting materials, *α*-bromoketones and *α*-bromaldehydes are available compounds. Selenourea and its derivatives are relatively expensive compounds and this circumstance can be considered a negative factor for the method, which uses the selenourea derivatives. The substituted selenoureas can be synthesized from secondary amines and organic isoselenocyanates. The latter compounds, in turn, serve as excellent starting materials for the synthesis of selenium/nitrogen heterocycles [134]. However, isoselenocyanates can be considered as not yet sufficiently accessible compounds. Although the classical method for obtaining organic isoselenocyanates is based on the reaction of isonitriles with elemental selenium, a number of other methods involve the use of highly toxic carbon diselenide [134].

In contrast, selenoamides are very accessible reagents. An effective and simple method that is commonly used to prepare selenoamides is the reaction of NaHSe with aryl or alkyl nitriles. Sodium hydroselenide can be easily prepared by the reaction of elemental selenium with sodium borohydride. Also, very convenient and reliable methods to obtain selenoamides are based on the reaction of nitriles with Woollins’ reagent and P_2_Se_5_.

Thus, although both approaches based on selenourea and selenamide derivatives lead to the target heterocyclic products in high yields, selenoamides are more accessible starting compounds compared to the selenourea derivatives and the approach with the use of selenoamides has an advantage in terms of starting compound availability.

The synthesis of ferrocene-bearing 1,3-selenazoles **15** demonstrates the broad scope of this approach (Scheme 8). Selenazoles **15** exhibit high antibacterial activity against *Escherichia coli*, *Staphylococcus aureus,* and *Pseudomonas aeruginosa* [53]. Using this approach, it is possible to combine two cycles in one molecules and to obtain bridged ring systems. Compounds (**19)** containing two 1,3-selenazole rings, separated by carbon bridges of different lengths, were obtained from dinitriles, P_2_Se_5_, and phenacyl bromide (Scheme 9) [50]. The synthesis of compound **17**, bearing the oxazole and selenazole rings, was also developed.

Selenazoles **34** and **36**, which contain two chlorine atoms or two iodine atoms linked to the selenium atom, exhibit antiviral activity against HIV-1 (Scheme 14) [58].

The convenient synthesis of a chromenone derivative **45**, containing the 1,3-selenazol ring, was developed by two approaches (Scheme 17) [61].

Some approaches to 1,3-selenazoles use eco-friendly reaction conditions and catalyst-free protocols for the synthesis of target products and meet the requirements of green chemistry (for example, the synthesis of 2-amino-1,3-selenazoles **70** through Hantzsch-type condensation (Scheme 27)) [71].

Compounds (**72)** show relatively high activity and low toxicity against the fungal strains *C. albicans* and *C. neoformans* [72].

According to research results, the products with F, NO_2_, CN, and MeO substituents and the cyclohexanone and 2-methylcyclohexanone derivatives **78** exhibit antifungal activity [73]. Compounds (**78)** with 3,4-dichlorophenyl and adamantanyl moieties show antibacterial activity [73].

The selenazoles **81** exhibit high antifungal activity against *Candida* spp. and antibacterial activity against Gram-positive bacteria. In addition, some compounds demonstrate significant anticonvulsant activity [74].

Compounds (**83)** were found to be active against Gram-positive bacteria, both pathogenic staphylococci and *Staphylococcus aureus* [75].

The selenazolyl-hydrazones **84** exhibit the properties of selective monoamine oxidase inhibitors with antioxidant and antiproliferative activities. These compounds are obtained by the reaction of *α*-bromocarbonyl derivatives and selenosemicarbazones [76].

Functionalized 1,3-selenazoles **86** were prepared based on selenosemicarbazide and 4-oxo-4*H*-chromene-3-carboxaldehyde. Compounds (**86)** show cytotoxic activity against Hep-G2, HCT-116, A549, and MCF-7 cancer cell lines [77].

Compounds **88** and **89** exhibit significant cytotoxic activities against two human cancer cell lines, hepatocarcinoma cells (Hep-G2) and prostate cancer cells (DU-145) [78].

The combination of the selenazole ring with sulfonamide moiety is favorable in respect to antitumor activity. Compounds (**140)** show potent inhibitory effects against breast cancer cell lines (MDA-MB-231) and human prostate cancer cells (PC3). In addition, these compounds show antibacterial activity [93,94].

Thus, significant progress has been made in the development and synthesis of 1,3-selenazole derivatives, and this area of research is currently being intensively developed. A number of promising compounds with high biological activity have been found that may find application in the future.

## Data Availability

Not applicable.
